# Activation of PI3K/AKT/mTOR Pathway Causes Drug Resistance in Breast Cancer

**DOI:** 10.3389/fphar.2021.628690

**Published:** 2021-03-15

**Authors:** Chao Dong, Jiao Wu, Yin Chen, Jianyun Nie, Ceshi Chen

**Affiliations:** ^1^ Department of the Second Medical Oncology, The 3rd Affiliated Hospital of Kunming Medical University, Yunnan Tumor Hospital, Kunming, China; ^2^ Department of Urology, Changhai Hospital, Navy Medical University, Shanghai, China; ^3^ Department of the Third Breast Surgery, The 3rd Affiliated Hospital of Kunming Medical University, Yunnan Tumor Hospital, Kunming, China; ^4^ Key Laboratory of Animal Models and Human Disease Mechanisms of Chinese Academy of Sciences and Yunnan Province, Kunming Institute of Zoology, Chinese Academy of Sciences, Kunming, China; ^5^ KIZ-CUHK Joint Laboratory of Bioresources and Molecular Research in Common Diseases, Kunming Institute of Zoology, Chinese Academy of Sciences, Kunming, China

**Keywords:** chemotherapy, targeted therapy, endocrine therapy, drug resistance, PI3K

## Abstract

Although chemotherapy, targeted therapy and endocrine therapy decrease rate of disease recurrence in most breast cancer patients, many patients exhibit acquired resistance. Hyperactivation of the PI3K/AKT/mTOR pathway is associated with drug resistance and cancer progression. Currently, a number of drugs targeting PI3K/AKT/mTOR are being investigated in clinical trials by combining them with standard therapies to overcome acquired resistance in breast cancer. In this review, we summarize the critical role of the PI3K/AKT/mTOR pathway in drug resistance, the development of PI3K/AKT/mTOR inhibitors, and strategies to overcome acquired resistance to standard therapies in breast cancer.

## Introduction

Breast cancer is the leading cause of cancer death in women around the world (Siegel and Miller, 2020). At the molecular level, breast cancer is a heterogeneous disease, divided into hormone/estrogen-receptor-positive (HR+/ER+), human epidermal growth factor receptor-2-positive (HER2+) and ER/PR/HER2 triple-negative breast cancer (TNBC) with corresponding treatment strategies according to molecular subtypes (Nagini, 2017). Common treatments include endocrine therapy (ET) for HR+ disease, HER2 targeted therapy for HER2+ disease, chemotherapy, and immunotherapy for TNBC patients as well as PARP inhibitors for BRCA-mutated TNBC patients. Acquired resistance leads to tumor relapse in breast cancer, which is associated with multiple but relatively independent mechanisms, including overexpression of breast cancer resistance protein (BCRP, also called ABCG2), modification of cell cycle checkpoints, inhibition of apoptosis, and activation of multiple signaling pathways (Kartal-Yandim et al., 2016).

The PI3K/AKT/mTOR pathway has emerged as a novel target for overcoming drug resistance in recent years (Keegan et al., 2018; Verret et al., 2019). Dysregulation of this pathway is closely related to tumor progression and resistance to standard therapies in breast cancer (Guerrero-Zotano et al., 2016). The PI3K/AKT/mTOR pathway is one of the most frequently activated pathways in several types of cancers (Alzahrani, 2019). This is also one of the most important reasons for intrinsic resistance. Several drugs against the PI3K/AKT/mTOR pathway are in clinical development. In this review, we summarize the current knowledge of the PI3K/AKT/mTOR pathway related to drug resistance in breast cancer and propose an effective drug development strategy.

## PI3K/AKT/mTOR Pathway and Their Inhibitors

### PI3K/AKT/mTOR Pathway

**FIGURE 1:**
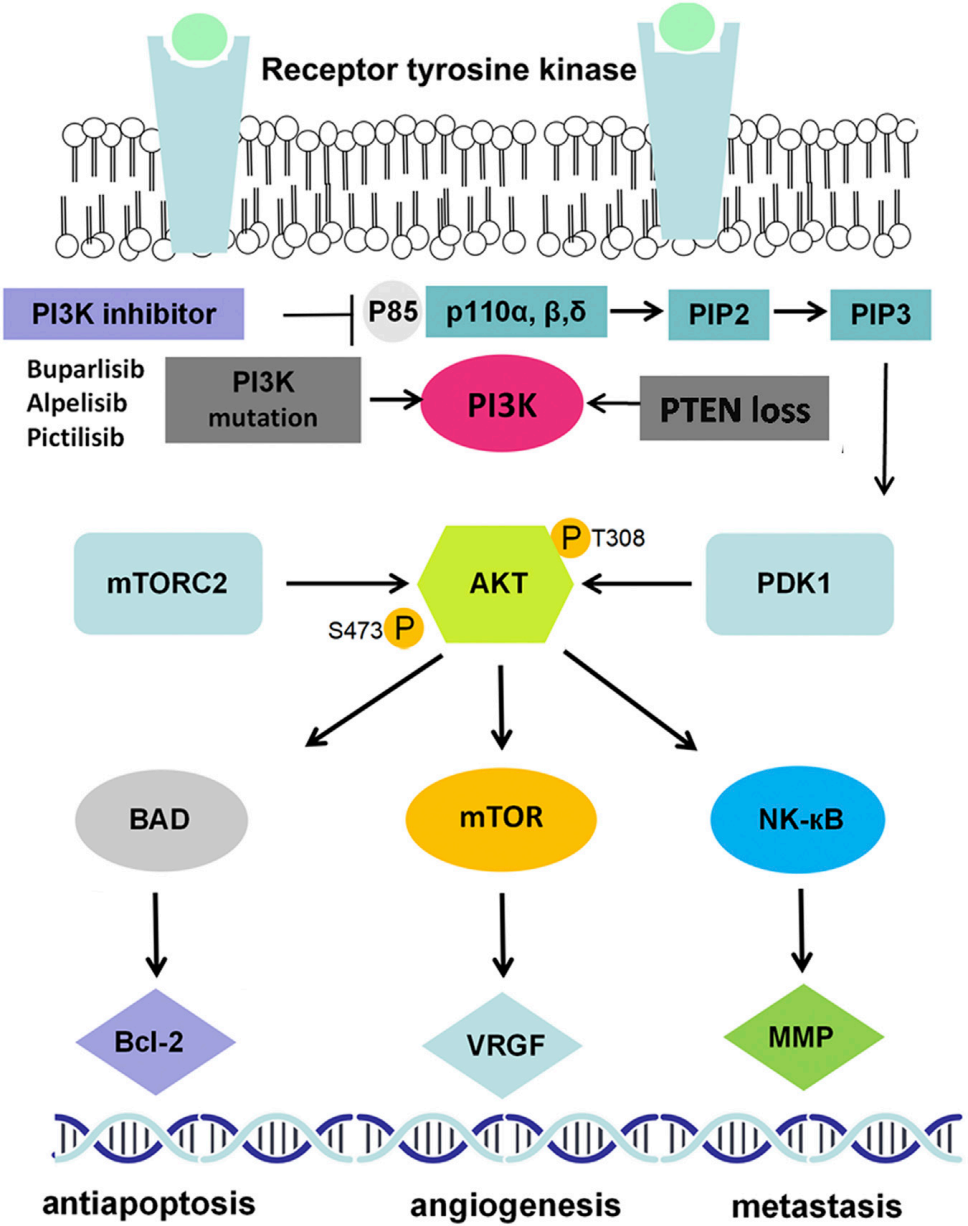
PI3K/AKT signaling pathway in breast cancer. EGF(R): epidermal growth factor (receptor); PI3K: phosphatidylinositol 3-kinase; PDK1/2: 3-phosphoinositide dependent kinase-1/2; mTOR: mammalian target of rapamycin; NF-κB: nuclear factor kappa-B; MMP9: matrix metallo protein 9; VEGF: vascular endothelial growth factor.

The PIK3CA gene is one of the most frequently mutated genes in breast cancer (Mukohara, 2015), which leads to PI3K activation and serves as a key determinant of standard anticancer therapy resistance (Liu, et al., 2019; Luo, et al., 2018; Qiu, et al., 2019). The PI3K protein belongs to a large family of lipid kinases, classified into three classes on the basis of different structures and functions. Among them, class I PI3Ks, the most fully studied heterodimers, consist of a p110 catalytic subunit and a p85 regulatory subunit, which play a predominant role in many cancers (Dornan and Burke, 2018). The PI3K protein has the dual activities of serine/threonine (Ser/Thr) kinase and phosphatidylinositol kinase and is activated in two ways: by interacting with various growth factor receptors, such as epithelial growth factor receptor (EGFR), vascular endothelial growth factor receptor (VEGFR) and fibroblast growth factor receptor (FGFR), and by recruiting an adaptor protein to promote the binding of p110 and p85 to activate PI3K. Activated PI3K can convert phosphatidylinositol 3,4-bisphosphate (PIP2) into 3,4,5-triphosphate (PIP3), PIP3 serves as the second messenger which can bind to phosphoinositide-dependent kinase-1 (PDK1) to phosphorylate AKT at Thr308. AKT can also be phosphorylated by mTORC2 at Ser473 (Figure 1). AKT is the key signal transduction protein that phosphorylates several substrates and downstream effectors, including mTOR, matrix metalloproteinase (MMP), cyclin-dependent kinases (CDKs), and VEGF (Liu et al., 2009; Cidado and Park, 2012) (Figure 1).

### PI3K/AKT/mTOR Inhibitors

To date, several PI3K inhibitors (PI3Ki), including alpelisib, idelalisib and copanlisib, have been approved by the Food and Drug Administration (FDA) (Janku et al., 2018) in many types of cancer. According to the targeted isoforms of class IA PI3Ks, PI3Ki can be classified into pan-PI3Ki, such as buparlisib (BKM120) and pictilisib (GDC-0941), isoform-specific PI3Ki, such as taselisib (GDC-0032) and alpelisib (BYL719), and dual mTOR/pan PI3Ki, such as BEZ235. BYL719 is the first oral PI3Ki that targets the p110α isoform selectively (IC_50_ = 4.6 nM) (Fritsch et al., 2014), and it was the first approved PI3K inhibitor combined with fulvestrant for patients with HR+/HER2− metastatic breast cancer (mBC) along with PIK3CA mutations that have progressed on or after endocrine-based regimens (Markham, 2019). Taselisib is another oral class I isoform-specific PI3K inhibitor, as it exhibits equal inhibition of p110α, p110γ and p110δ (Ndubaku et al., 2013). Buparlisib, an oral pan-PI3K inhibitor, targets all four isoforms of class I PI3K (Hu et al., 2015). Pictilisib (GDC-0941) is another oral pan-PI3K inhibitor that shows equipotent efficiency in inhibiting the p110α and p110δ isoforms *in vitro* (Martín et al., 2017). Based on previous results of clinical trials, compared to pan-PI3K inhibitors, these isoform-specific PI3K inhibitors show improved tolerability and increased anticancer efficacy (Krop et al., 2016; Baselga et al., 2017; André et al., 2019); thus, more selective PI3K inhibitors are expected in the future.

According to the structure and the binding site, AKT inhibitors (AKTi) can be divided into PH domain inhibitors: allosteric inhibitors and ATP competitive inhibitors. Several PH domain inhibitors and allosteric inhibitors were terminated in the preclinical stage due to poor pharmacokinetic properties and heavy toxicity effects. The current research hotspot is ATP competitive inhibitors such as ipatasertib (GDC-0068) and capivasertib (AZD-5363). As a downstream molecule of the PI3K/AKT pathway, mTOR inhibitors can inhibit breast cancer cell growth. Everolimus was the first approved mTOR inhibitor for HR+/HER2− breast cancer and showed an improved PFS in several clinical trials (Bachelot et al., 2012; Yardley et al., 2013), whereas another mTOR inhibitor, temsirolimus, showed only moderate activity in HR+ breast cancer patients with age ≤ 65 years, but this randomized phase III trial was terminated for futility as total PFS was not improved by temsirolimus plus letrozole as first-line therapy, so the best suitable population for temsirolimus needs to be further identified (Wolff et al., 2013). Detailed information on these PI3K/AKT/mTOR inhibitors is listed in Table 1.

**TABLE 1:** Summary clinical RCT trials of PI3K/AKT/mTOR inhibitors in breast cancer.

Drug	Target	Study	Phase	Patients population	Therapy line	Regimen	Outcome
Buparlisib (BKM120), oral	Class I PI3K-	BELLE-2 (NCT01610284)	III	Postmenopausal, HR(+)/HER2(−), AI-resistant locally advanced or mBC	Second-line or later	Buparlisib + fulvestrant	mPFS: 6.9 vs. 5.0 m (HR 0.78, *p* = 0.00021)
						Placebo + fulvestrant	mPFS: 6.8 vs. 4.0 m (HR 0.76, *p* = 0.014) in PIK3CA-mutant subset
	p110α						mOS: 33.2 vs. 30.4 m (HR 0.87, *p* = 0.045)
	p110β	BELLE-3 (NCT01633060)	III	Postmenopausal, HR(+)/HER2(−), mTOR inhibitor-resistant, locally advanced or mBC	Second-line or later	Buparlisib + fulvestrant	mPFS: 3.9 vs. 1.8 m (HR 0.67, *p* = 0.0003)
	p110δ					Placebo + fulvestrant	
	p110γ	NeoPHOEBE (NCT01816594)	II	HER2(+) primary BC	Neoadjuvant	Buparlisib + trastuzumab + paclitaxel	ORR: 69 vs. 33% (*p* = 0.053)
						Placebo + trastuzumab + paclitaxel	pCR: 32 vs. 40% (*p* = 0.811)
		BELLE-4 (NCT01572727)	II/III	HER2(−) primary with locally advanced or mBC	First-line	Buparlisib + paclitaxel	mPFS: 8.0 vs. 9.2 m (HR 1.18)
						Placebo + paclitaxel	mPFS: 9.1 vs. 9.2 m (HR 1.17) in PIK3CA-mutant subset
Pictilisib (GDC-0941), oral	Class I PI3K-	PEGGY (NCT01740336)	II	HR(+)/HER2(−) locally advanced or mBC	Second-line or later	Pictilisib + paclitaxel	mPFS: 8.2 vs. 7.8 m (HR 0.95, *p* = 0.83)
	p110α					Placebo + paclitaxel	mPFS: 7.3 vs. 5.8 m (HR 1.06, *p* = 0.88) in PIK3CA-mutant subset
	p110δ	FERGI (NCT01437566)	II	Postmenopausal, ER (+)/HER2(−), AI-resistant advanced or mBC	Second-line or later	Pictilisib + fulvestrant	mPFS: 6.6 vs. 5.1 m (HR 0.74, *p* = 0.096)
						Placebo + fulvestrant	mPFS: 6.5 vs. 5.1 m (HR 0.74, *p* = 0.268) in PIK3CA-mutant subset
Alpelisib (BYL719), oral	Class I PI3K-	SOLAR-1 (NCT02437318)	III	PIK3CA-mutated, previously received endocrine therapy, HR (+)/HER2(−) advanced BC	First or second-line	Alpelisib + fulvestrant	mPFS: 11.0 vs. 5.7 m (HR 0.65, *p* = 0.00065) in PIK3CA-mutant subset
	p110α					Placebo + fulvestrant	ORR: 26.6 vs. 12.8% in PIK3CA-mutant subset
	p110α-H1047R	NEO-ORB (NCT01923168)	II	Postmenopausal, HR (+)/HER2(−) early stage BC	Neoadjuvant	Alpelisib + letrozole	ORR: 63 vs. 61% in PI3K wide-type subset
	p110α-E545K					Placebo + letrozole	ORR: 43 vs. 45% in PI3K-mutant subset
Taselisib (GDC-0032), oral	Class I PI3K-	LORELEI (NCT02273973)	II	HR (+)/HER2(−) operable early stage BC	Neoadjuvant	Taselisib + letrozole	ORR: 50 vs. 39% (OR 1.55, *p* = 0.049)
	p110δ					Placebo + letrozole	ORR: 56 vs. 38% (OR 2.03, *p* = 0.033) in PIK3CA-mutant subset
	p110α	SANDPIPER (NCT02340221)	III	HR(+)/HER2(−), AI resistant locally advanced or mBC	Second-line or later	Taselisib + fulvestrant	mPFS: 7.4 vs. 5.4 m (HR 0.70, *p* = 0.0037)
	p110γ					Placebo + fulvestrant	
Capivasertib (AZD-5363), oral	Akt1 Akt2 Akt3	FAKTION (NCT01992952)	II	HR(+)/HER2(−), AI-resistant advanced BC	Second-line or later	Capivasertib + fulvestrant	mPFS: 10.3 vs. 4.8 m (HR 0.58, *p* = 0.0035)
						Placebo + fulvestrant	mOS: 26.0 vs. 20.0 m (HR 0.59, *p* = 0.071)
Ipatasertib (GDC-0068), oral	Akt1 Akt2 Akt3	LOTUS (NCT02162719)	II	Primary locally advanced or mTNBC	First-line	Ipatasertib + paclitaxel	mPFS: 6.2 vs. 4.9 m (HR 0.60, *p* = 0.037)
						Placebo + paclitaxel	mPFS: 6.2 vs. 3.7 m (HR 0.59, *p* = 0.18) in PTEN-low cohort
Everolimus, oral	mTOR1	BOLERO-2 (NCT00863655)	III	HR (+)/HER2(−), AI-resistant and postmenopausal advanced BC	Second-line or later	Everolimus + exemestrane	mPFS: 10.6 vs. 4.1 m (HR 0.36, *p* < 0.001)
						Placebo + exemestrane	ORR: 7 vs. 0.4% (*p* < 0.001)
		MANTA (NCT02216786)	II	HR (+), postmenopausal and AI-resistant locally advanced or mBC	Second-line or later	Everolimus + fulvestrant	mPFS: 12.3 vs. 5.4 m (HR 0.63, *p* = 0.01)
						Fulvestrant	
		PrE0102 (NCT01797120)	II	HR (+)/HER2(−), AI-resistant and postmenopausal mBC	Second-line or later	Everolimus + fulvestrant	mPFS: 10.3 vs. 5.1 m (HR 0.61, *p* = 0.02)
						Placebo + fulvestrant	ORR: 18.2 vs. 12.3% (*p* = 0.47)
		BOLERO-1 (NCT00876395)	III	HER2(+), primary advanced BC	First-line	Everolimus + trastuzumab	mPFS: 14.9 vs. 14.5 m (HR 0.89, *p* = 0.12)
						Placebo + trastuzumab	mPFS: 20.3 vs. 13.1 m (HR 0.66, *p* = 0.0049) in HR (-) tumors
		BOLERO-3 (NCT01007942)	III	HER2(+), taxane-pretreated and trastuzumab-resistant advanced BC	Second-line or later	Everolimus + trastuzumab + vinorelbine	mPFS: 7.0 vs. 5.8 m (HR 0.78, *p* = 0.0067)
						Placebo + trastuzumab+ vinorelbine	
Temsirolimus (CCI-779), intravenous	mTOR	HORIZON (NCT00083993)	III	HR(+), postmenopausal, AI-naïve advanced BC	First-line	Temsirolimus + letrozole	mPFS: 8.9 vs. 9.0 m (HR 0.90, *p* = 0.25)
						Placebo + letrozole	mPFS: 9.0 vs. 5.6 m (HR 0.70, *p* = 0.009) in age ≤ 65 years subgroup

AI, aromatase inhibitor; mBC, metastatic breast cancer.

In addition, activation of the PI3K/AKT pathway can upregulate multidrug resistance-associated protein-1 (MRP1), ABCG2 and P-glycoprotein (P-gp, also called ABCB1) expression, which could be involved in multidrug resistance (Tazzari et al., 2007; Wang et al., 2016a). Dysregulation of the PI3K/AKT pathway has an impact on resistance to chemotherapy, endocrine therapy, HER2− targeted therapy, PARP inhibitors, and immunotherapy in breast cancer, and blockage of this pathway can enhance drug sensitivity and reverse resistance (Miller et al., 2011a; Hu et al., 2015; Pernas and Tolaney, 2019). Overall, accumulated evidence suggests that PI3K/AKT inhibitors could combine with other anticancer therapies to overcome drug resistance in breast cancer.

## PI3K/AKT/mTOR Pathway Induces Drug Resistance in Breast Cancer

### PI3K/AKT/mTOR Pathway in Endocrine Therapy-Resistant Breast Cancer

Several types of endocrine drugs, including selective ER modulators (tamoxifen), aromatase inhibitors (AIs, such as letrozole and exemestane), and selective ER degraders (fulvestrant), are the most important therapies for ER+ breast cancer. In addition to the well-known efficiency of these treatments, approximately 50% of patients develop disease progression due to endocrine resistance (Chen et al., 2020a). Therefore, it is important to improve the survival and prognosis of breast cancer by exploring endocrine resistance mechanisms to develop new treatment strategies. At present, the known mechanisms of endocrine resistance are mainly related to alteration of ER structure and function, including downregulation or loss of ER expression, ER gene mutations, posttranslational modification of ER, abnormality of ER coactivators, and the interaction between ER and various intracellular signal molecules, such as HER2, EGFR, PI3K/AKT/mTOR and MAPK/ERK (Zhao and Ramaswamy, 2014).

**FIGURE 2:**
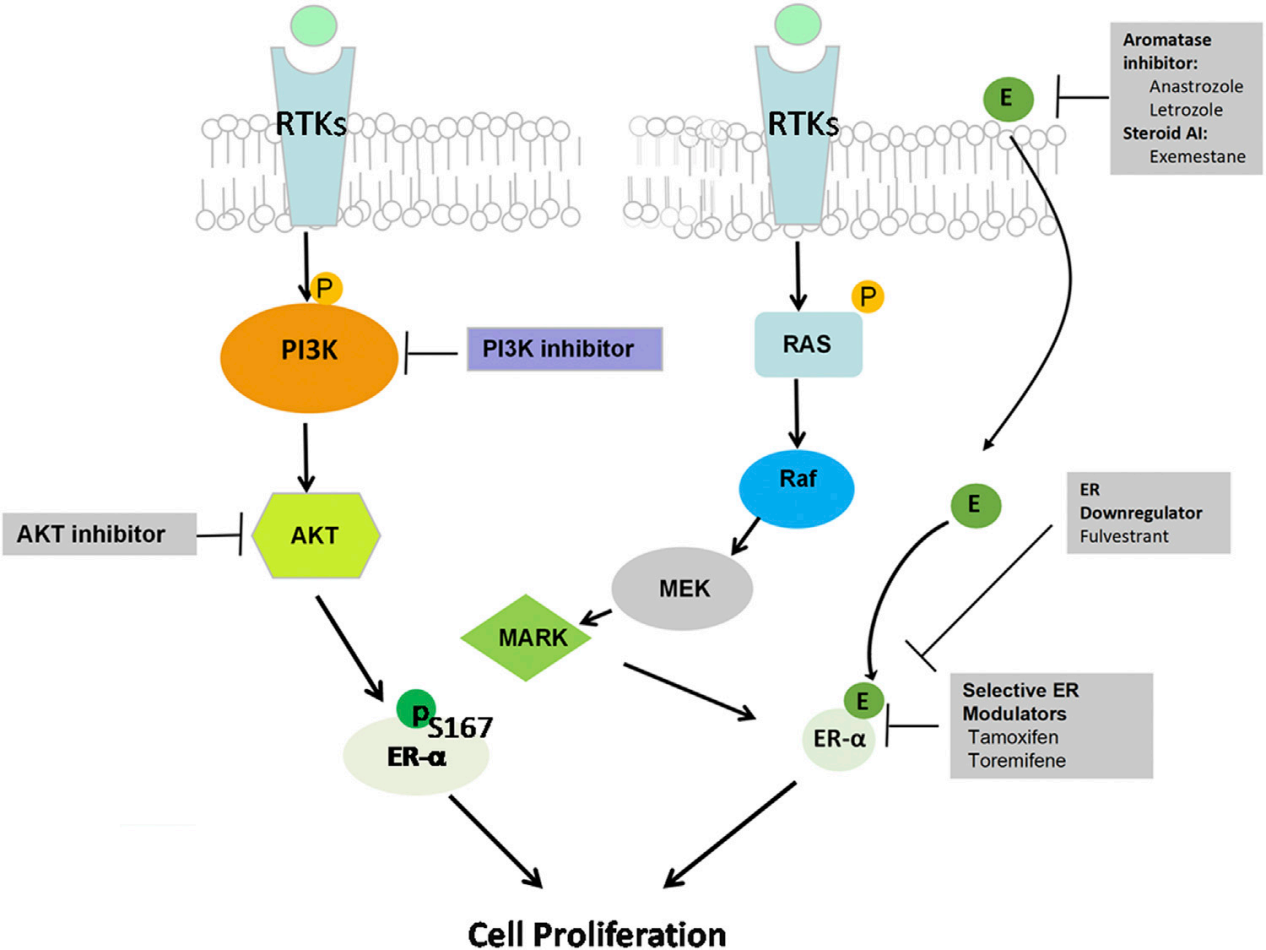
PI3K/AKT pathway and hormone therapy resistance. The interaction between ER signaling and the RTK pathway is considered a major mechanism of resistance to hormone therapy. Upregulation of both the PI3K/AKT/mTOR and RAS/RAF/MAPK pathways activates the ERα-independent pathway in the absence of estrogen though extranuclear ER signaling, thus causing estrogen-regulated gene activation that ultimately promotes cancer cell survival and leads to hormone therapy resistance.

Accumulating data indicate the role of PI3K activation in endocrine resistance, and the development of PI3K inhibitors is an attractive strategy to reverse endocrine resistance (Yap et al., 2015). Preclinical data support that PI3K and AKT can phosphorylate ERα at Ser167 to activate ERα independently in the absence of estrogen, so the interaction between ER and PI3K/AKT/mTOR pathway hyperactivation causes breast cancer cells to lose sensitivity to endocrine therapy (Campbell et al., 2001) (Figure 2). Upregulation of the PI3K/AKT pathway plays a critical role in endocrine treatment resistance through extranuclear ER signaling (Mukohara, 2015). During the progression of endocrine resistance, PI3K mainly activates mTOR substrate p70S6 kinase in MCF-7 and MDA-MB-361 cells after long-term estrogen deprivation (Miller, et al., 2010). In addition, PIK3CA mutations or PTEN loss can induce estrogen-independent growth of breast cancer cells (Miller, et al., 2011b). PIK3CA mutations are detected in close to 50% of ER+ breast cancer patients, which contribute to endocrine resistance (Miller, et al., 2011c; Huang et al., 2019). Furthermore, the mechanism of resistance involves upregulation of receptor tyrosine kinases (RTKs), which leads to enhanced activation of the downstream PI3K/AKT signaling pathway (Mansouri et al., 2018) (Figure 2). PI3K/AKT inhibition combined with different types of endocrine drugs, such as tamoxifen, AIs, and fulvestrant, is a new strategy for ER+ breast cancer treatment (Sabine, et al., 2014; Hanamura and Hayashi, 2018). Miller et al. reported that endocrine-resistant cell proliferation depends on the PI3K pathway and becomes extremely sensitive when inhibiting this pathway (Miller et al., 2010). Then, in an *in vitro* study, Sanchez et al. demonstrated that PI3K inhibitors showed potent anti-proliferative activity when combined with endocrine therapy in ER+ MCF-7 breast cells (Sanchez et al., 2011). The ER degrader fulvestrant combined with a p110α and p110β isoform inhibitor induced apoptosis and suppressed PTEN-deficient xenograft tumor growth by inhibiting PI3K downstream substrates, indicating that it is an effective strategy to overcome endocrine resistance in the luminal B subtype of breast cancer due to reduced PTEN levels (Hosford et al., 2017). Therefore, these results implied that PI3K/AKT/mTOR inhibition can restore ER expression levels and activity and improve sensitivity to endocrine therapy.

Until now, clinical trials using drugs targeting PI3K/AKT/mTOR plus hormone therapy have shown promising results in HR+/HER2− mBC. These results are summarized in Table 1. The combination of endocrine therapy with most PI3K inhibitors, such as buparlisib in the BELLE-2 study and the PI3Kα inhibitors alpelisib and taselisib in the SOLAR-1 and SANDPIPER studies, respectively, has demonstrated potent anticancer effects in HR+/HER2− and AI-resistant breast cancer (Baselga et al., 2017; Baselga et al., 2018; André et al., 2019), although the pan-PI3K inhibitor pictilisib in the FERGI study showed limited efficacy in AI-resistant advanced or mBC. In addition, AKT inhibitor of capivasertib combined with fulvestrant (FAKTION study) also showed an improved PFS and OS in AI-resistant advanced BC (Jones et al., 2020). Furthermore, letrozole combined with the PI3Kα inhibitor taselisib (LORELEI study) but not alpelisib (NEO-ORB study) showed a synergetic anticancer effect as neoadjuvant therapy in early stage breast cancer. Moreover, inhibition of mTOR, a downstream target of the PI3K pathway, has also been clinically shown to enhance the anticancer effect of endocrine therapy with AIs in HR+ advanced BC(Baselga et al., 2012). Consistently, patients with AI-resistant advanced BC have consistently been shown to obtain significant clinical benefits from everolimus in several phase II-III clinical trials that combined everolimus with endocrine therapy, including BOLERO-2 (NCT00863655), MANTA (NCT02216786) and PrE0102 (NCT01797120). Therefore, based on these promising preclinical and clinical data, several ongoing studies of PI3K inhibitors in combination with hormonal therapy as a strategy to overcome hormonal therapy-resistant breast cancer are being conducted, including the BYLieve study (NCT03056755) and the SAFIR study (NCT02734615, NCT03386162, NCT01870505).

### PI3K/AKT/mTOR Pathway in HER2-Targeted Therapy-Resistant Breast Cancer

**FIGURE 3:**
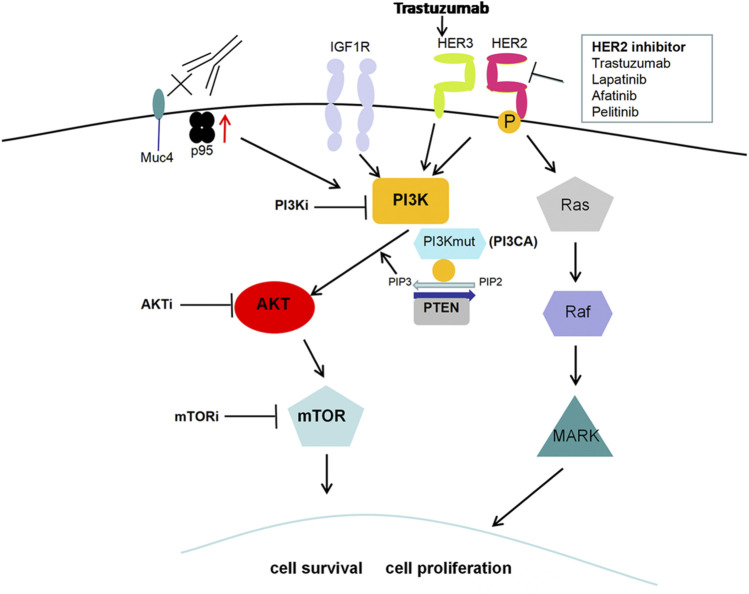
PI3K/AKT pathway and HER2-targeted therapy resistance. High expression of the HER2 truncated mutant (p95-HER2), the most common form of HER2 structural change, is related to HER2-targeted therapy resistance through activating the PI3K/AKT pathway. Trastuzumab treatment increases HER3 expression and leads to PI3K/AKT activation along with subsequent resistance to anti-HER2 therapy. Additionally, PI3KCA mutations, PTEN loss or both, also contribute to PI3K activation and then promote cell proliferation along with resistance to HER2-targeted therapy.

In addition to HER2 monoclonal antibody drugs, including trastuzumab and pertuzumab, several small-molecule tyrosine kinase inhibitors, such as lapatinib, neratinib and pyrotinib, and an antibody-drug conjugate (T-DM1) have emerged for HER2+ breast cancer treatment in recent years. Anti-HER2 therapy has greatly improved the survival and prognosis of HER2+ breast cancer, but acquired resistance still occurs (Nagini, 2017). Further study of the mechanism of HER2-targeted therapy resistance is important. Attention has been given to the abnormal activation of the PI3K/AKT/mTOR signaling pathway, which is closely associated with anti-HER2 therapy resistance (Pernas and Tolaney, 2019). The common form of HER2 structural change is the HER2 truncated mutant (p95-HER2). High expression of p95-HER2 indicates poor prognosis and is related to the drug resistance of anti-HER2 therapy through activating the PI3K/AKT pathway (Arribas et al., 2011). HER3 is overexpressed in breast cancer and can potently activate the downstream PI3K/AKT pathway, which promotes cancer cell survival and leads to drug resistance (Dey et al., 2015). It was reported that trastuzumab also increased HER3 expression, thus leading to trastuzumab resistance (Li et al., 2018). Additionally, PI3KCA mutations, PTEN loss or both, leading to PI3K activation, are related to tumor progression and reduced survival rate in breast cancer, which has an adverse effect on the therapeutic effect of trastuzumab (Razis et al., 2011) (Figure 3). PIK3CA somatic mutations mainly include two “hot spots,” H1047R or H1047L in exon 20 and E542K or E545K in exon 9. E545K and H1047R are prevalent in breast cancer, which leads to lapatinib resistance (Eichhorn et al., 2008). Mutation of the PIK3CA gene promotes anti-HER2 resistance via the catalytic subunit p110α but not p110β. The combination of alpelisib and lapatinib effectively reduced breast cancer cell growth in genetic models driven by HER2 activation and PTEN loss (Wang et al., 2016b). However, in recent years, some clinical studies have suggested that a single PI3KCA mutation or PTEN loss cannot accurately predict trastuzumab resistance, and more molecular markers need to be combined (Pogue-Geile et al., 2015; Stern et al., 2015).

A number of preclinical studies suggest that PI3K/AKT/mTOR inhibition can overcome anti-HER2 therapy resistance. PI3K inhibition by BAY 80-6946 has been confirmed to resensitize trastuzumab and/or lapatinib-resistant cells to HER2-targeted therapies (Elster et al., 2015). Similar results suggest that alpelisib plus anti-HER2 Ab largely inhibit breast cancer tumor growth (Zhang et al., 2016). The pan-PI3K inhibitor NVP-BKM120 significantly suppresses PIK3CA-mutant tumor xenograft growth when combined with HER2-targeted therapy (Maira et al., 2012). In addition, PI3K inhibitors and BEZ235 (dual PI3K/mTOR inhibitor) suppress trastuzumab-resistant breast cancer cell proliferation by inhibiting the PI3K/AKT/mTOR pathway (Kataoka et al., 2010; O’Brien et al., 2014). BEZ235 can also overcome resistance to lapatinib and EGFR/HER2 inhibitors in PIK3CA-activated or PTEN-deficient breast cancer cell lines (Brünner-Kubath et al., 2011). Furthermore, mTOR inhibition combined with lapatinib leads to higher synergistic tumor response rates in trastuzumab-resistant xenografts (Gayle et al., 2012). It was reported that lapatinib-resistant HER2+ breast cancer cells increased the expression levels of survivin and c-IAP2, two inhibitors of apoptosis (IAPs), and treatment with the mTOR kinase inhibitor AZD8055 reversed expression of IAPs and overcame lapatinib resistance in lapatinib-resistant cells (Brady et al., 2015). Moreover, targeted therapy against HER2 and the PI3K/mTOR pathway induce apoptosis and promote trastuzumab-resistant xenograft shrinkage (Vuylsteke et al., 2016; Kim et al., 2017).

Based on the results of these preclinical studies, many clinical trials were conducted as described in Table 1, NeoPHOEBE (NCT01816594) showed that the HER2-targeted therapy trastuzumab was enhanced when the pan-PI3K inhibitor buparlisib was combined in HER2+ primary breast cancer (Loibl et al., 2017). In addition, some other clinical trials assessing the therapeutic effect of everolimus plus HER2-targeted therapy have also shown optimistic results, including BOLERO-3 (NCT01007942) and BOLERO-1 (NCT00876395) studies (André et al., 2014; Hurvitz et al., 2015). Currently, several phase I clinical trials of PI3K selective inhibitors in combination with HER2-targeted therapy designed to further evaluate these findings are ongoing. Firstly, a phase I trial (NCT02038010) combining PI3Kα inhibitor alpelisib and trastuzumab-MCC-DM1 (T-DM1) in HER2+ mBC patients who have progressed from prior treatment with trastuzumab plus taxane-based chemotherapy is currently being conducted. Another new PI3Kα selective inhibitor, MEN1611, is being tested in a phase Ib trial (NCT03767335) to evaluate the effect of MEN1611 plus trastuzumab combined with or without fulvestrant in PIK3CA-mutated patients who were diagnosed with HER2+, advanced or mBC and have progressed after HER2 targeted therapy. Furthermore, a Phase I, open-label study (NCT02167854) is ongoing to understand the best dose of alpelisib combined with LJM716 (anti-HER3 Ab) and trastuzumab in HER2+ mBC. In addition to PI3Kα inhibitor, the PI3Kδ isoform inhibitor copanlisib combined with trastuzumab, a phase Ib/II trial (NCT04108858), is being tested in HER2-targeted therapy-resistant breast cancer. Overall, PI3K/AKT/mTOR inhibition is emerging as a potential therapeutic strategy to overcome HER2-targeted therapy resistance.

### PI3K/AKT/mTOR Pathway in Chemotherapy-Resistant Breast Cancer

**FIGURE 4:**
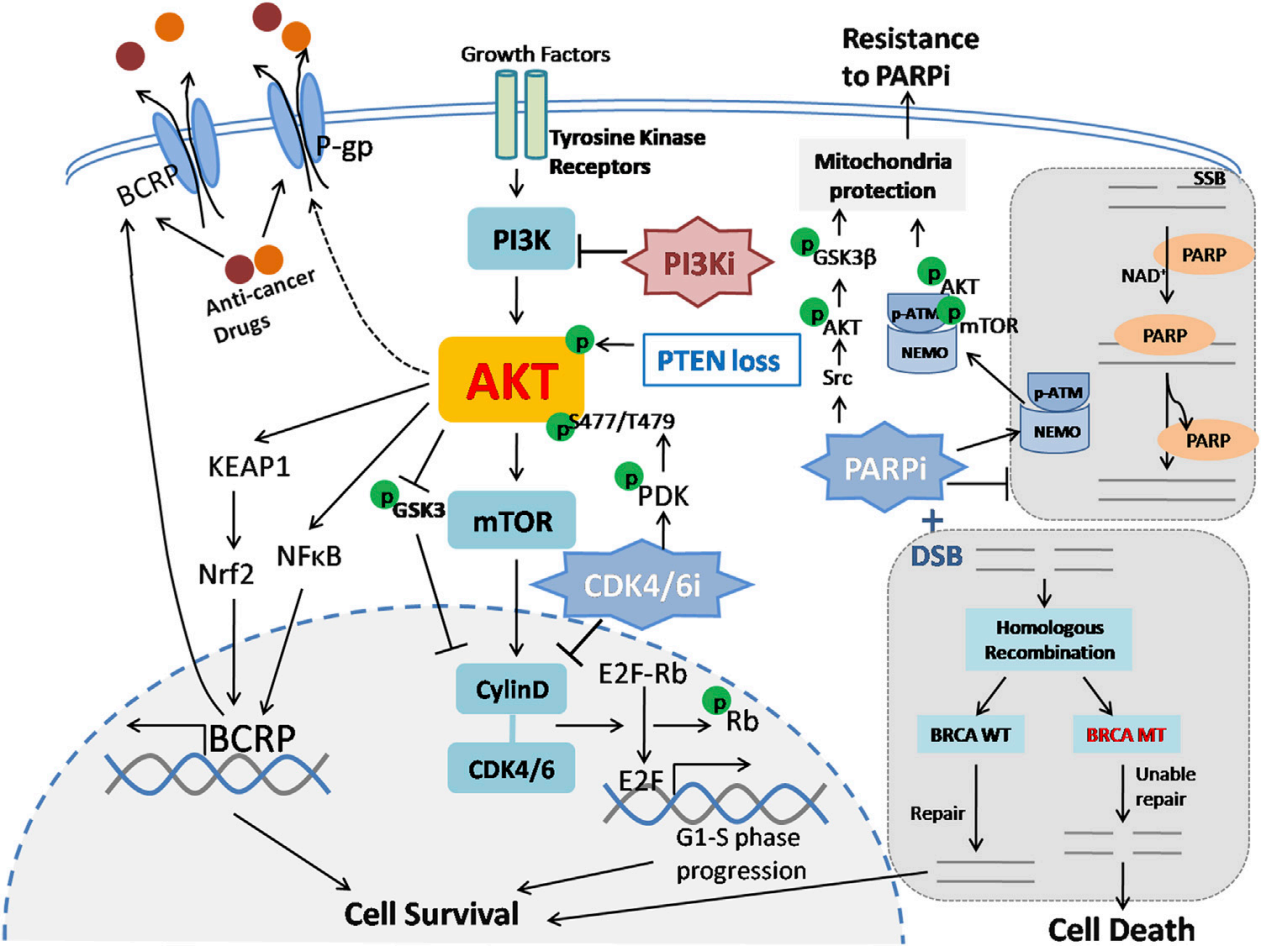
PI3K/AKT pathway is related to chemotherapy, CDK4/6 and PARP inhibitor resistance. 1) ABC transporters, including BCRP and P-gp, enhance the efflux of chemotherapeutic drugs and cause chemoresistance. PI3K/AKT promotes BCRP transcription via the KEAP1-Nrf2 axis and NF-κB pathway and induces P-gp expression with an uncharacterized mechanism. Subsequently, drug outflow is increased through ABC transporters, which ultimately results in resistance to chemotherapy in breast cancer. ABC: ATP-binding cassette; KEAP1: Kelch-like ECH-associated protein 1; Nrf2: nuclear factorerythroid-derived 2. 2) The CDK4/6 complex promotes Rb phosphorylation and dissociates Rb from E2F transcription factors, thus promoting cell cycle G1-S phase progression and cell survival. CDK4/CDK6 inhibitors can block this process and inhibit tumor cell proliferation. However, CDK4/CDK6 inhibition therapy leads to PI3K/AKT pathway activation via phosphorylation of AKT at the S477/T479 site by PDK1. Additionally, activated AKT can also upregulate cyclin D expression by inhibiting GSK3 phosphorylation. As a result, breast cancer cells gradually become resistant to CDK4/CDK6 inhibitors. CDK4/CDK6: Cyclin D/cyclin-dependent kinase 4/6; Rb: retinoblastoma protein. 3) PARP is activated by DNA damage and catalyzes the covalent coupling of branched chains of ADP-ribose units, thus promoting DNA repair and maintaining genomic stability. In BRCA-mutated cells, double-strand broken DNA is unable to be repaired via homologous recombination; such cells fail to repair and ultimately exhibit synthetic lethality in the presence of DNA damage and PARP inhibitors. Suppression of PARP1 activity induces AKT and its downstream target GSK3β phosphorylation via Src and facilitates cytoplasmic translocation of the pATM-NEMO complex and thereby forms a cytoprotective signalosome, both of which contribute to mitochondrial protection under oxidative stress conditions and lead to acquired resistance to PARP inhibitors. PARP: Poly (ADP-ribose) polymerases; SSB: Single-strand break; DSB: Double-strand break.

Chemotherapy also plays an important role during breast cancer treatment, especially in TNBC. However, chemotherapy failure often occurs because of the acquisition of anticancer resistance and acquired drug resistance (Olgen, 2018). Unfortunately, the underlying mechanisms of chemoresistance in breast cancer have not yet been clearly clarified. The biological causes of drug resistance are attributed to diverse molecular mechanisms, including reduced drug accumulation, metabolic detoxification, anti-apoptosis, autophagy, and other mechanisms (Ji et al., 2019). At present, studies have demonstrated that PI3K/AKT promotes chemotherapy resistance through multidrug resistance-related proteins and anti-apoptosis effects (Zhang et al., 2020). It was reported that PI3K/AKT increases the outflow of chemotherapeutic drugs through the ATP-binding cassette (ABC) transporter superfamily, thus causing and maintaining the multidrug resistance of tumor cells (Kathawala et al., 2015) (Figure 4). In a number of MDR cancer cells, BCRP and P-gp proteins are overexpressed, which mediates the extracellular release of taxanes and anthracyclines (Kathawala et al., 2015). Additionally, AKT phosphorylation has been shown to be positively associated with cell viability, migration and apoptosis in breast cancer, which may induce chemoresistance (Gao et al., 2019). Therefore, blocking PI3K/AKT can restore the sensitivity of cancer cells to chemotherapy drugs and thus overcome drug resistance.

Several preclinical studies have indicated that PI3K/AKT/mTOR inhibitors have an important effect on reversing the chemoresistance of breast cancer. The PI3K inhibitor wortmannin nearly completely blocked cell proliferation and resistance to doxorubicin in MCF-7/ADR (ADR: Adriamycin resistance) breast cancer cells (Tsou et al., 2015). Similarly, our previous study showed that the inhibition of PI3K by econazole significantly sensitized MDA-MB-231 and MCF-7 cells to adriamycin *in vitro* and *in vivo* (Dong et al., 2020). In addition, the pan-PI3K inhibitor BKM120 showed a potent antitumor ability in both sensitive and MDR breast cancer cells by inhibiting the PI3K/AKT/NF-κB signaling pathway, and the combination of BKM120 with doxorubicin showed a synergistic effect (Hu et al., 2015). Moreover, doxorubicin combined with the pan-PI3K inhibitor buparlisib also revealed a strong synergistic anti-proliferative effect (Hu et al., 2015). Furthermore, it was reported that 3-methyladenine (3-MA) blocks the PI3K/AKT/mTOR pathway and reverses MDR by inhibiting agent-efflux transporters (Zou et al., 2014). Consistently, another study reported that BEZ235 combined with tunicamycin could promote autophagy and apoptosis and then enhance chemosensitivity in MCF-7 cells by suppressing the PI3K/AKT pathway (Zhong et al., 2017).

In the clinic, two randomized controlled trial (RCT) studies, PEGGY (NCT01740336) (Vuylsteke et al., 2016) and BELLE-4 (NCT01572727) (Martín et al., 2017), were conducted to evaluate the efficiency of adding PI3K inhibitors to paclitaxel in advanced BC. However, median PFS was only slightly prolonged but without significance in both studies, even in the PIK3CA-mutant subset. In addition, in the phase 2 LOTUS study (NCT02162719), inoperable, locally advanced or metastatic primary TNBC patients were recruited and randomly assigned to two groups treated with paclitaxel plus AKT inhibitor ipatasertib or placebo. The median PFS was improved in the ipatasertib arm (6.2 months vs 4.9 months, *p* = 0.037), and in the subset with low PTEN expression, the median PFS was also increased from 3.7 months to 6.2 months by ipatasertib treatment (*p* = 0.18), which is the first clinical result supporting AKT-targeted therapy for TNBC (Kim et al., 2017). These results are listed in Table 1. At present, several clinical trials, including the SAFIR study (NCT02379247), BEECH study (NCT01625286) and IPATunity130 (NCT03337724), are ongoing to assess the effect of the PI3Kα inhibitor alpelisib or AKT inhibitor of AZD5363 or ipasertib plus paclitaxel in advanced BC or mBC. Overall, the potential role of PI3K/AKT inhibitors in overcoming chemoresistance is an attractive prospect in breast cancer therapy.

### PI3K/AKT/mTOR Pathway in PARP Inhibitor-Resistant Breast Cancer

In recent years, the poly-ADP-ribose polymerase inhibitors (PARPis) olaparib and talazoparib were approved by the FDA for germline BRCA1 and BRCA2 mutation (gBRCA1/2+) mBC (Hoy, 2018; Tompa, 2018). Olaparib presents a significant clinical benefit, and a series of clinical trials showed that compared to standard chemotherapy, olaparib improved PFS in patients with gBRCA-mutated HER2-mBC, with a manageable toxicity profile (Griguolo et al., 2018). However, since acquired resistance is gradually emerging, there is an urgent need to develop strategies to overcome resistance to PARP inhibitors in breast cancer.

The mechanism of PARPi resistance is complex and includes drug efflux, homologous recombination repair restoration, dysfunction of the cell cycle and signal transduction (Bitler et al., 2017). Preclinical data have indicated that PI3K/AKT/mTOR activation is related to PARPi resistance. PARP inhibitors can result in PI3K/AKT pathway activation via Src under oxidative conditions (Veres et al., 2003; Tapodi et al., 2005; Veres et al., 2004). Another study found that PARP suppression inhibits the PARylation of ATM and promotes an ATM-NEMO interaction, subsequently facilitating cytoplasmic translocation of this complex and forming a p-ATM-NEMO-Akt-mTOR cytoprotective signalosome (Tapodi et al., 2019), indicating that using combined inhibitors targeting the PI3K/AKT/mTOR pathway and PARPi may potentially obtain a better clinical benefit (Figure 4). In TNBC patient-derived primary tumor xenografts, dual inhibition of PI3K and PARP has been shown to significantly reduce the growth of tumors (Ibrahim et al., 2012). Similarly, the combination of BKM120 and olaparib dramatically extended the doubling time of tumors in an MMTV-Cre: Brca1(f/f): Trp53 (+/−) breast cancer mouse model (Juvekar et al., 2012). In addition, using rapamycin to inhibit S6 phosphorylation, an mTOR downstream effector, can restore the PARPi sensitivity of resistant BC cells, and the combination of rapamycin and PARPi can obviously suppress BRCA1-deficient tumor growth *in vivo* (Sun et al., 2014). Furthermore, it has been reported that compared to PARPi alone, the combination of PARP and PI3K inhibitors creates a synergetic antitumor ability to inhibit BRCA1-deficient breast tumor growth *in vitro* and *in vivo* (Zhang et al., 2019).

Currently, there are two clinical trials to determine the combined effect of PARPi and PI3K/AKT inhibitors in TNBC. One is a phase I study (NCT01623349) to assess olaparib combined with PI3K inhibitors BYL719 or BKM120 in patients diagnosed with mTNBC that have progressed from prior treatments including at least one cytotoxic agent. Another is a phase I/II study (NCT02208375), in which olaparib plus AZD 2014 (mTOR inhibitor) or AZD5363 (AKT inhibitor) regimens are being assessed in several types of cancers, including TNBC. Therefore, the combination of PARPi with PI3K/AKT/mTOR inhibitors may potentially increase clinical benefit and enhance the response to PARPi.

### PI3K/AKT/mTOR Pathway in CDK4/6-Inhibited or CDK4/6-Resistant Breast Cancer

In addition to PARPi, CDK4/6 inhibitors, such as ribociclib, abemaciclib and palbociclib, have been approved for HR+/HER2− mBC treatment, and improved survival was observed by combining with endocrine therapy (Murphy, 2019). However, acquired resistance is inescapable, and the management of primary and acquired resistance to CDK4/6 inhibitors is urgent.

Until now, several cell cycle-related mechanisms have been determined as possible reasons for resistance, including RB1 or FAT1 loss mutations, CDK6 and CCNE1 overexpression or amplification, FGFR alterations and PI3K/mTOR-mediated CDK2 activation (Xi and Ma, 2020). PI3K/mTOR pathway activation is a potential mechanism for both primary and acquired resistance to CDK4/6 inhibitors, and the synergetic effect of PI3K inhibition post-CDK4/6 inhibitor treatment has been confirmed in several studies (Zhang et al., 2016; Jansen et al., 2017). Under ribociclib treatment, a kinome-wide siRNA knockdown screen was conducted in MCF-7 cells and showed that PDK1 was identified as a key modifier of ribociclib sensitivity, which is a PI3K pathway component required for AKT activation (Jansen et al., 2017) (Figure 4). Combining PI3K inhibitors with CDK4/6i has been confirmed to have a synergistic effect to overcome intrinsic and adaptive resistance in multiple studies (Vora et al., 2014; Herrera-Abreu et al., 2016). CDK4/6 and PI3K inhibitors together have been shown to induce breast cancer cell apoptosis *in vitro* and suppress tumor growth in patient-derived tumor xenograft models (Herrera-Abreu et al., 2016). Additionally, mTORC1/2 inhibition has been shown to inhibit Rb phosphorylation, cyclin D1 expression and E2F-mediated transcription in CDK4/6i-resistant cells (Michaloglou et al., 2018). In this regard, the BYLieve (NCT03056755) study is being conducted to evaluate the effect of alpelisib in the subset of HR+/HER2− recurrent BC patients with PIK3CA mutations who have progressed on prior CDK4/6 inhibitor treatment. Another trial (NCT02088684) examined the anticancer effect of the triplet combination regimen of ribociclib, fulvestrant and buparlisib or alpelisib compared to the combination of ribociclib with fulvestrant. Several trials are assessing the efficacy of the triplet regimen in breast cancer, including NCT02871791, NCT02732119, NCT02088684, NCT02057133, NCT02389842, NCT02684032, and NCT01857193. In addition, several other ongoing studies (NCT02871791, NCT01857193, NCT02732119) are being conducted to test triplet regimens by combining CDK4/6i with exemestane and everolimus.

### PI3K/AKT/mTOR Pathway in Immunotherapy-Resistant Breast Cancer

In the last decade, cancer immunotherapy has obtained marked clinical efficacy during cancer therapy. In breast cancer, several PD-1/PD-L1 inhibitors, such as pembrolizumab, avelumab and atezolizumab, are in clinical trials (Emens, 2018), although only atezolizumab as a first-line therapy has been approved by the FDA to treat patients with advanced metastatic TNBC expressing PD-L1 (Soare and Soare, 2019). In several preclinical studies, the PI3K/AKT/mTOR pathway has been preliminarily proven to be a critical pathway for sustaining the function of immunosuppressive Tregs. On the one hand, pan-AKT inhibitors such as wortmannin, IC87114 or MK-2206 enhanced CD8+ T cell infiltration in the tumor microenvironment (Abu-Eid et al., 2014). Similarly, the functions of CD8+ T cells were sustained under P110δ inhibition (Patton et al., 2006). On the other hand, the differentiation process of memory T cells seems to be affected by the PI3K/AKT/mTOR network. Using rapamycin to inhibit mTOR significantly enhanced memory T cell differentiation (Araki et al., 2009). Another study demonstrated that AKT inhibition also potently accelerated the differentiation of memory T cells (van der Waart et al., 2014). AKT can inhibit TCR/LEF/β-catenin and FOXO transcription, which are required for the function of T cell memory (Lazarevic et al., 2013). Specifically, AKT inhibition can led to a favorable immune profile in the breast tumor microenvironment, including an increased density of CD3+CD8+ cells and better expression of interferon genes, providing a rationale for using AKT inhibition plus immunotherapy combination (Marks et al., 2020). Correspondingly, the PI3Kβ inhibitor AZD8186 enhanced anticancer efficacy by combining with anti-PD1 Ab in PTEN-deficient xenografts (Owusu-Brackett et al., 2020). Combining nanoscale PI3K-targeting supramolecular therapeutics with anti-PD-L1/PD-1 Ab exhibited a synergetic anticancer effect in breast cancer *in vivo* (Kulkarni et al., 2016). Therefore, these preclinical data suggest that the combination of PI3K/AKT/mTOR inhibitors may be effective in overcoming resistance to immune checkpoint blockade therapy in breast cancer. However, further clinical studies need to be conducted to understand the safety and efficacy of these combinations.

### PI3K/AKT/mTOR Pathway in Radio-Resistant Breast Cancer

Aberrant upregulation and activation of PI3K/AKT/mTOR pathway is also correlated with resistance radiotherapy. A number of preclinical studies indicated that inhibition of PI3K/AKT/mTOR pathway can sensitize different types of cancer cells, including non-small cell lung cancer, breast cancer, nasopharyngeal carcinoma, oral squamous, and head and neck cancer, to radiotherapy (Yu et al., 2017; Chen et al., 2020b; Chen et al., 2020c; Glorieux et al., 2020; Schötz et al., 2020; Wanigasooriya et al., 2020). In breast cancer, IL-6 pre-treatment followed by SIRT1 activation by SRT1720 combined with dual PI3K/mTOR inhibitor NVP-BEZ235 obviously increased sensitivity of the cancer stem cells to radiation, so that late stage breast cancer cells therapeutic effect was effectively improved (Fatehi et al., 2018; Masoumi et al., 2020). Another dual PI3K/mTOR inhibitor PI103 could significantly induce prolongation of γH2AX foci and also effectively sensitized SKBR3 cells to radiotherapy (No et al., 2009). Accordingly, further pre-clinical studies should be focused on dual PI3K/mTOR inhibitors. However, no relevant clinical trials have been conducted until now, thus early stage clinical studies are needed to understand the feasibility and efficacy of these dual PI3K/mTOR inhibitors in combination with radiotherapy in cancer patients.

## Potential Predictive Biomarkers of Response or Resistance to PI3K Inhibitors

### PIK3CA Mutation Predicts the Response to PI3K Inhibitors

PI3K inhibitors show different activities in a range of different populations, indicating that there is a need for patient selection. PIK3CA mutation may serve as a potential predictive marker, which is of considerable interest in trials so far (Mollon et al., 2020). Notably, the combination of fulvestrant and alpelisib has shown synergistic antitumor activity against ER+/PIK3CA-mutated advanced BC (André et al., 2019). PIK3CA mutation-induced endocrine therapy resistance is of great interest since PIK3CA is frequently mutated in luminal tumors (Network, 2012). Therefore, PIK3CA mutations represent potential new therapeutic targets to overcome resistance and may be an appropriate therapeutic effect response predictor of PI3K inhibitors (Brandão et al., 2019).

PIK3CA somatic mutations mainly include two “hot spots,” which have presented a gain-of-function transforming capacity in PI3K signaling (Ching-Shian Leong et al., 2008). The synergistic effect of PI3K inhibitors has been shown to be much more dramatic in PIK3CA-mutated breast cancer cells than in PIK3CA WT cells, and E545K mutants in exon 9 have shown a relatively poor response to PI3K inhibitor (Liu et al., 2018). In the PALOMA-3 randomized phase III trial, decreased PIK3CA variants in patients were detected at the end of treatment compared to initial treatment (26.7 vs. 19.0%). Clonal evolution was frequently found during treatment by exome sequencing of plasma DNA, suggesting abundant subclonal complexity in breast cancer patients who had progressed on previous endocrine therapy (O’Leary et al., 2018). Therefore, it is rational to observe tumor clonal evolution prior to treatment and after disease progression to identify more predictive biomarkers.

However, there is a slight controversial correlation between the PIK3CA genotype and response to the combination PI3K inhibitor and endocrine therapy. In the BELLE-2 III trial as previously mentioned, those patients with PIK3CA mutations obtained an obviously improved PFS from the buparlisib plus fulvestrant therapy arm (Baselga et al., 2017). In contrast, a significant PFS benefit was not observed from the pan-PI3K inhibitor pictilisib plus fulvestrant regimen in the FERGI study (Krop et al., 2016). On the one hand, compared to PIK3CA WT tumors, PIK3CA mutant tumors tended to be resistant to these therapies. Previous trials have failed to confirm that PIK3CA mutations possess predictive value for RFS from tamoxifen treatment (Beelen et al., 2014). On the other hand, compared with patients with PIK3CA WT status, breast cancer patients with PIK3CA mutations treated with AIs showed an improvement in time to progression (Ramirez-Ardila et al., 2013). Studies have also shown that PI3K is hyperactivated in long-term estrogen-deprived cells and that fulvestrant plus BKM120 induced suppression of ER+ xenografts (Miller et al., 2011). Nevertheless, most of the current phase III PI3K inhibitor clinical trials are investigating endocrine therapy-resistant advanced BC by combining PI3Ki with endocrine therapy, which has a synergistic effect and may help reverse endocrine therapy resistance.

### PTEN Loss Predicts the Response to PI3K Inhibitors

Activation of the PI3K/AKT/mTOR pathway is attributed to several factors, such as PIK3CA mutation, PTEN loss, RAS/MEK/ERK, ER or HER2 pathway activation, FGFR1/2 amplification, KRAS and TP53 mutations. PTEN, a key tumor suppressor gene, negatively regulates the PI3K/AKT pathway, and PTEN loss can lead to out-of-control PI3K signal transduction, which results in breast cancer cell growth (Li et al., 1997). It has been reported that nearly 40–50% of breast tumors harbor heterozygous PTEN loss, whereas functional PTEN loss because of mutations has been detected in 5–10% of breast tumors, and the frameshift is the most common mechanism (Coughlin et al., 2010; Bazzichetto et al., 2019). Preclinical data indicate that breast cancer cells with PTEN expression loss are more sensitive to PI3K/AKT inhibitors (O’Brien et al., 2010). In patients who had progressed on prior alpelisib treatment, *de novo* PTEN expression loss has been reported in all metastatic lesions; accordingly, PTEN-null xenografts derived from these patients showed resistance to alpelisib (Juric et al., 2015). In addition, some PTEN-deficient tumors are dependent on PI3Kβ signaling because depletion of PIK3CA has no impact on cell growth in three PTEN-deficient cancer cell lines, whereas inhibition of the PIK3CB isoform, responsible for encoding p110β, resulted in tumor growth suppression *in vitro* and *in vivo* (Wee et al., 2008). Furthermore, in the phase 2 LOTUS clinical trial (NCT02162719), inoperable, locally advanced or metastatic primary TNBC patients were recruited and treated with paclitaxel plus either ipatasertib (AKT inhibitor) or placebo, and in the low PTEN expression subset, median PFS was prolonged by ipatasertib as previously described (Kim et al., 2017).

To date, only the PIK3CA mutation has been proven to be a therapeutic efficiency predictive marker for PI3Kα inhibitor treatment in advanced BC. For PTEN, more evidence from preclinical and clinical studies is needed to further investigate its predictive value for the efficiency of PI3Ki treatment in breast cancer patients.

### Other Potential Biomarkers

In addition to two critical predictive biomarkers (PIK3CA mutation and PTEN loss) as described above, there are several potential biomarkers are under exploring. It was reported that patients with early metabolic response by ^18F^FDG-PET/CT often showed an increased benefit from buparlisib and letrozole combination treatment in a phase Ib clinical trial (Mayer et al., 2014), that is to say, a decreased metabolism in tumors predict better response to PI3Ki. Additionally, results from OPPORTUNE trial demonstrated that patients with progesterone receptor-negative status present a better response from pictilisib (Schmid et al., 2016). What’s more, several possible mechanisms of PI3Ki resistance have been uncovered in recent years. Firstly, overexpression of ribosomal S6 kinases RPS6KA2 (RSK3) and RPS6KA6 (RSK4) promoted breast cancer growth upon PI3K inhibition treatment, which can be reversed by addition of MEK- or RSK-specific inhibitors (Serra et al., 2013). Secondly, it was reported that PI3Ki-resistant cell lines can be resensitized by AKTi MK-2206 (Vora et al., 2014). Moreover, PI3K inhibition can result in hyperglycaemia as PI3K mediates cellular responses to insulin, which leads to an increasing release of insulin in a feedback manner and re-activate PI3K pathway in tumor models in mice, thus ultimately causing PI3Ki resistance (Hopkins et al., 2018). High insulin levels may be a potential biomarker for predicting resistance to PI3Ki, which could be partially prevented by ketogenic diet and sodium-glucose cotransporter inhibitors (Hopkins et al., 2018). In a phase Ib trial, patients with TP53 mutations, KRAS, or FGFR1/2 amplification did not obtain clinical benefit from letrozole/alpelisib treatment (Mayer et al., 2017), thus these mutations or amplification may also serve as potential biomarkers to PI3Ki resistance.

## Summary

Breast cancer drug resistance is a complex phenomenon that results in treatment failure and disease progression. Inhibitors of the PI3K/AKT/mTOR pathway combined with existing treatment methods can act to overcome drug resistance in different subtypes of breast cancer. In this review, our perspective is that PI3K/AKT/mTOR pathway activation is related to conventional therapy resistance in breast cancer, including endocrine therapy, HER2-targeted therapy, CDK4/6 and PARP inhibitors, chemotherapy and immunotherapy. However, the poor selectivity of the inhibitors is an important factor leading to toxicity. The pan-PI3K inhibitors have shown mild results and significant toxicity. In contrast, the more selective PI3K inhibitors, such as alpelisib, have shown promising results, particularly in patients with PIK3CA mutations. Overall, PI3K/AKT/mTOR pathway inhibitors show the potential ability to restore sensitivity to standard treatments, which is an attractive strategy in overcoming resistance during breast cancer treatment.

A number of issues still should be focused on in the near future. Initially, more highly selective PI3K inhibitors should be developed to obtain better tolerability and anticancer efficiency. Second, further studies should be conducted to identify reliable biomarkers to predict response and resistance as well as avoid unnecessary side effects. Third, more optimized combination regimens need to be investigated for the best synergistic effect. Additionally, PI3K/AKT/mTOR inhibitor resistance should also be investigated. What’s more, intrinsic resistance is inevitable besides acquired resistance since PI3K/AKT/mTOR pathway is basally activated in multiple solid tumors, including breast cancer. Therefore, most importantly, more effective clinical trials should be conducted to adequately confirm or support the preclinical results so that the survival time and quality of life of resistant breast cancer patients may be largely further improved.

## References

[B1] Abu-Eid R. Samara R. N. Ozbun L. Abdalla M. Y. Berzofsky J. A. Friedman K. M. (2014). Selective inhibition of regulatory T cells by targeting the PI3K-Akt pathway. Cancer Immunol. Res. 2, 1080–1089. 10.1158/2326-6066.cir-14-0095 25080445 PMC4221428

[B2] Alzahrani A. S. (2019). PI3K/Akt/mTOR inhibitors in cancer: at the bench and bedside. Semin. Cancer Biol. 59, 125–132. 10.1016/j.semcancer.2019.07.009 31323288

[B3] André F. Ciruelos E. Rubovszky G. Campone M. Loibl S. Rugo H. S. (2019). Alpelisib for PIK3CA-mutated, hormone receptor-positive advanced breast cancer. N. Engl. J. Med. 380, 1929–1940. 10.1056/NEJMoa1813904 31091374

[B4] André F. O’Regan R. Ozguroglu M. Toi M. Xu B. Jerusalem G. (2014). Everolimus for women with trastuzumab-resistant, HER2-positive, advanced breast cancer (BOLERO-3): a randomised, double-blind, placebo-controlled phase 3 trial. Lancet Oncol. 15, 580–591. 10.1016/s1470-2045(14)70138-x 24742739

[B5] Araki K. Turner A. P. Shaffer V. O. Gangappa S. Keller S. A. Bachmann M. F. (2009). mTOR regulates memory CD8 T-cell differentiation. Nature 460, 108–112. 10.1038/nature08155 19543266 PMC2710807

[B6] Arribas J. Baselga J. Pedersen K. Parra-Palau J. L. (2011). p95HER2 and breast cancer. Cancer Res. 71, 1515–1519. 10.1158/0008-5472.can-10-3795 21343397

[B7] Bachelot T. Bourgier C. Cropet C. Ray-Coquard I. Ferrero J. M. Freyer G. (2012). Randomized phase II trial of everolimus in combination with tamoxifen in patients with hormone receptor-positive, human epidermal growth factor receptor 2-negative metastatic breast cancer with prior exposure to aromatase inhibitors: a GINECO study. J. Clin. Oncol. 30, 2718–2724. 10.1200/jco.2011.39.0708 22565002

[B8] Baselga J. Campone M. Piccart M. Burris H. A. 3rd Rugo H. S. Sahmoud T. (2012). Everolimus in postmenopausal hormone-receptor-positive advanced breast cancer. N. Engl. J. Med. 366, 520–529. 10.1056/NEJMoa1109653 22149876 PMC5705195

[B9] Baselga J. Im S. A. Iwata H. Cortés J. De Laurentiis M. Jiang Z. (2017). Buparlisib plus fulvestrant versus placebo plus fulvestrant in postmenopausal, hormone receptor-positive, HER2-negative, advanced breast cancer (BELLE-2): a randomised, double-blind, placebo-controlled, phase 3 trial. Lancet Oncol. 18, 904–916. 10.1016/s1470-2045(17)30376-5 28576675 PMC5549667

[B10] Baselga J. Dent S. F. Cortés J. Im Y. H. Diéras V. Harbeck N. (2018). Phase III study of taselisib (GDC0032) + fulvestrant (FULV) v FULV in patients (pts) with estrogen receptor (ER)-positive, PIK3CA-mutant (MUT), locally advanced or metastatic breast cancer (MBC): primary analysis from SANDPIPER. J. Clin. Oncol. 36, LBA1006. 10.1007/s10549-018-4697-y

[B11] Bazzichetto C. Conciatori F. Pallocca M. Falcone I. Fanciulli M. Cognetti F. (2019). PTEN as a prognostic/predictive biomarker in cancer: an unfulfilled promise? Cancers 11, 435. 10.3390/cancers11040435 PMC6520939 30925702

[B12] Beelen K. Opdam M. Severson T. M. Koornstra R. H. Vincent A. D. Wesseling J. (2014). PIK3CA mutations, phosphatase and tensin homolog, human epidermal growth factor receptor 2, and insulin-like growth factor 1 receptor and adjuvant tamoxifen resistance in postmenopausal breast cancer patients. Breast Cancer Res. 16, R13. 10.1186/bcr3606 24467828 PMC3978618

[B13] Bitler B. G. Watson Z. L. Wheeler L. J. Behbakht K. (2017). PARP inhibitors: clinical utility and possibilities of overcoming resistance. Gynecol. Oncol. 147, 695–704. 10.1016/j.ygyno.2017.10.003 29037806 PMC5698126

[B14] Brady S. W. Zhang J. Tsai M. H. Yu D. (2015). PI3K-independent mTOR activation promotes lapatinib resistance and IAP expression that can be effectively reversed by mTOR and Hsp90 inhibition. Cancer Biol. Ther. 16, 402–411. 10.1080/15384047.2014.1002693 25692408 PMC4623386

[B15] Brandão M. Caparica R. Eiger D. de Azambuja E. (2019). Biomarkers of response and resistance to PI3K inhibitors in estrogen receptor-positive breast cancer patients and combination therapies involving PI3K inhibitors. Ann. Oncol. 30 (Suppl. 10), x27–x42. 10.1093/annonc/mdz280 PMC6923785 31859350

[B16] Brünner-Kubath C. Shabbir W. Saferding V. Wagner R. Singer C. F. Valent P. (2011). The PI3 kinase/mTOR blocker NVP-BEZ235 overrides resistance against irreversible ErbB inhibitors in breast cancer cells. Breast Cancer Res. Treat. 129, 387–400. 10.1007/s10549-010-1232-1 21046231

[B17] Campbell R. A. Bhat-Nakshatri P. Patel N. M. Constantinidou D. Ali S. Nakshatri H. (2001). Phosphatidylinositol 3-kinase/AKT-mediated activation of estrogen receptor alpha: a new model for anti-estrogen resistance. J. Biol. Chem. 276, 9817–9824. 10.1074/jbc.M010840200 11139588

[B18] Chen K. Quan J. Yang J. Chen Z. (2020a). The potential markers of endocrine resistance among HR+/HER2+ breast cancer patients. Clin. Transl Oncol. 22, 576–584. 10.1007/s12094-019-02163-2 31209793

[B19] Chen K. Shang Z. Dai A. L. Dai P. L. (2020b). Novel PI3K/Akt/mTOR pathway inhibitors plus radiotherapy: strategy for non-small cell lung cancer with mutant RAS gene. Life Sci. 255, 117816. 10.1016/j.lfs.2020.117816 32454155

[B20] Chen Q. Zheng W. Zhu L. Yao D. Wang C. Song Y. (2020c). ANXA6 contributes to radioresistance by promoting autophagy via inhibiting the PI3K/AKT/mTOR signaling pathway in nasopharyngeal carcinoma. Front. Cell Dev. Biol. 8, 232. 10.3389/fcell.2020.00232 32373608 PMC7176914

[B21] Ching-Shian Leong V. Jabal M. F. Leong P. P. Abdullah M. A. Gul Y. A. Seow H. F. (2008). PIK3CA gene mutations in breast carcinoma in Malaysian patients. Cancer Genet. Cytogenet. 187, 74–79. 10.1016/j.cancergencyto.2008.07.005 19027487

[B22] Cidado J. Park B. H. (2012). Targeting the PI3K/Akt/mTOR pathway for breast cancer therapy. J. Mammary Gland Biol. Neoplasia 17, 205–216. 10.1007/s10911-012-9264-2 22865098 PMC3724399

[B23] Coughlin C. M. Johnston D. S. Strahs A. Burczynski M. E. Bacus S. Hill J. (2010). Approaches and limitations of phosphatidylinositol-3-kinase pathway activation status as a predictive biomarker in the clinical development of targeted therapy. Breast Cancer Res. Treat. 124, 1–11. 10.1007/s10549-010-1108-4 20803067

[B24] Dey N. Williams C. Leyland-Jones B. De P. (2015). A critical role for HER3 in HER2-amplified and non-amplified breast cancers: function of a kinase-dead RTK. Am. J. Transl Res. 7, 733–750. 26064441 PMC4455348

[B25] Dong C. Chen Y. Ma J. Yang R. Li H. Liu R. (2020). Econazole nitrate reversed the resistance of breast cancer cells to Adriamycin through inhibiting the PI3K/AKT signaling pathway. Am. J. Cancer Res. 10, 263–274. 10.7150/ijbs.32625 32064166 PMC7017736

[B26] Dornan G. L. Burke J. E. (2018). Molecular mechanisms of human disease mediated by oncogenic and primary immunodeficiency mutations in class IA phosphoinositide 3-kinases. Front. Immunol. 9, 575. 10.3389/fimmu.2018.00575 29616047 PMC5868324

[B27] Eichhorn P. J. Gili M. Scaltriti M. Serra V. Guzman M. Nijkamp W. (2008). Phosphatidylinositol 3-kinase hyperactivation results in lapatinib resistance that is reversed by the mTOR/phosphatidylinositol 3-kinase inhibitor NVP-BEZ235. Cancer Res. 68, 9221–9230. 10.1158/0008-5472.can-08-1740 19010894 PMC2587064

[B28] Elster N. Cremona M. Morgan C. Toomey S. Carr A. O’Grady A. (2015). A preclinical evaluation of the PI3K alpha/delta dominant inhibitor BAY 80-6946 in HER2-positive breast cancer models with acquired resistance to the HER2-targeted therapies trastuzumab and lapatinib. Breast Cancer Res. Treat. 149, 373–383. 10.1007/s10549-014-3239-5 25528022

[B29] Emens L. A. (2018). Breast cancer immunotherapy: facts and hopes. Clin. Cancer Res. 24, 511–520. 10.1158/1078-0432.ccr-16-3001 28801472 PMC5796849

[B30] Fatehi D. Soltani A. Ghatrehsamani M. (2018). SRT1720, a potential sensitizer for radiotherapy and cytotoxicity effects of NVB-BEZ235 in metastatic breast cancer cells. Mol. Biol. Rep. 214, 889–895. 10.1007/s11033-019-05114-w 29653746

[B31] Fritsch C. Huang A. Chatenay-Rivauday C. Schnell C. Reddy A. Liu M. (2014). Characterization of the novel and specific PI3Kα inhibitor NVP-BYL719 and development of the patient stratification strategy for clinical trials. Mol. Cancer Ther. 13, 1117–1129. 10.1158/1535-7163.mct-13-0865 24608574

[B32] Gao C. Yuan X. Jiang Z. Gan D. Ding L. Sun Y. (2019). Regulation of AKT phosphorylation by GSK3β and PTEN to control chemoresistance in breast cancer. Breast Cancer Res. Treat. 176, 291–301. 10.1007/s10549-019-05239-3 31006103

[B33] Gayle S. S. Arnold S. L. O’Regan R. M. Nahta R. (2012). Pharmacologic inhibition of mTOR improves lapatinib sensitivity in HER2-overexpressing breast cancer cells with primary trastuzumab resistance. Anticancer Agents Med. Chem. 12, 151–162. 10.2174/187152012799015002 22043997 PMC3288300

[B34] Glorieux M. Dok R. Nuyts S. (2020). The influence of PI3K inhibition on the radiotherapy response of head and neck cancer cells. Sci. Rep. 10, 16208. 10.1038/s41598-020-73249-z 33004905 PMC7529775

[B35] Griguolo G. Dieci M. V. Guarneri V. Conte P. (2018). Olaparib for the treatment of breast cancer. Expert Rev. Anticancer Ther. 18, 519–530. 10.1080/14737140.2018.1458613 29582690

[B36] Guerrero-Zotano A. Mayer I. A. Arteaga C. L. (2016). PI3K/AKT/mTOR: role in breast cancer progression, drug resistance, and treatment. Cancer Metastasis Rev. 35, 515–524. 10.1007/s10555-016-9637-x 27896521

[B37] Hanamura T. Hayashi S. I. (2018). Overcoming aromatase inhibitor resistance in breast cancer: possible mechanisms and clinical applications. Breast Cancer 25, 379–391. 10.1007/s12282-017-0772-1 28389808

[B38] Herrera-Abreu M. T. Palafox M. Asghar U. Rivas M. A. Cutts R. J. Garcia-Murillas I. (2016). Early adaptation and acquired resistance to CDK4/6 inhibition in estrogen receptor-positive breast cancer. Cancer Res. 76, 2301–2313. 10.1158/0008-5472.can-15-0728 27020857 PMC5426059

[B39] Hopkins B. D. Pauli C. Du X. Wang D. G. Li X. Wu D. (2018). Suppression of insulin feedback enhances the efficacy of PI3K inhibitors. Nature 560, 499–503. 10.1038/s41586-018-0343-4 30051890 PMC6197057

[B40] Hosford S. R. Dillon L. M. Bouley S. J. Rosati R. Yang W. Chen V. S. (2017). Combined inhibition of both p110α and p110β isoforms of phosphatidylinositol 3-kinase is required for sustained therapeutic effect in PTEN-deficient, ER(+) breast cancer. Clin. Cancer Res. 23, 2795–2805. 10.1158/1078-0432.ccr-15-2764 27903677 PMC5449270

[B41] Hoy S. M. (2018). Talazoparib: first global approval. Drugs 78, 1939–1946. 10.1007/s40265-018-1026-z 30506138

[B42] Hu Y. Guo R. Wei J. Zhou Y. Ji W. Liu J. (2015). Effects of PI3K inhibitor NVP-BKM120 on overcoming drug resistance and eliminating cancer stem cells in human breast cancer cells. Cell Death Dis. 6, e2020. 10.1038/cddis.2015.363 26673665 PMC4720896

[B43] Huang D. Tang L. Yang F. Jin J. Guan X. (2019). PIK3CA mutations contribute to fulvestrant resistance in ER-positive breast cancer. Am. J. Transl Res. 11, 6055–6065. 31632573 PMC6789267

[B44] Hurvitz S. A. Andre F. Jiang Z. Shao Z. Mano M. S. Neciosup S. P. (2015). Combination of everolimus with trastuzumab plus paclitaxel as first-line treatment for patients with HER2-positive advanced breast cancer (BOLERO-1): a phase 3, randomised, double-blind, multicentre trial. Lancet Oncol. 16, 816–829. 10.1016/s1470-2045(15)00051-0 26092818

[B45] Ibrahim Y. H. García-García C. Serra V. He L. Torres-Lockhart K. Prat A. (2012). PI3K inhibition impairs BRCA1/2 expression and sensitizes BRCA-proficient triple-negative breast cancer to PARP inhibition. Cancer Discov. 2, 1036–1047. 10.1158/2159-8290.cd-11-0348 22915752 PMC5125254

[B46] Janku F. Yap T. A. Meric-Bernstam F. (2018). Targeting the PI3K pathway in cancer: are we making headway? Nat. Rev. Clin. Oncol. 15, 273–291. 10.1038/nrclinonc.2018.28 29508857

[B47] Jansen V. M. Bhola N. E. Bauer J. A. Formisano L. Lee K. M. Hutchinson K. E. (2017). Kinome-wide RNA interference screen reveals a role for PDK1 in acquired resistance to CDK4/6 inhibition in ER-positive breast cancer. Cancer Res. 77, 2488–2499. 10.1158/0008-5472.can-16-2653 28249908 PMC5421398

[B48] Ji X. Lu Y. Tian H. Meng X. Wei M. Cho W. C. (2019). Chemoresistance mechanisms of breast cancer and their countermeasures. Biomed. Pharmacother. 114, 108800. 10.1016/j.biopha.2019.108800 30921705

[B49] Jones R. H. Casbard A. Carucci M. Cox C. Butler R. Alchami F. (2020). Fulvestrant plus capivasertib versus placebo after relapse or progression on an aromatase inhibitor in metastatic, oestrogen receptor-positive breast cancer (FAKTION): a multicentre, randomised, controlled, phase 2 trial. Lancet Oncol. 21, 345–357. 10.1016/s1470-2045(19)30817-4 32035020 PMC7052734

[B50] Juric D. Castel P. Griffith M. Griffith O. L. Won H. H. Ellis H. (2015). Convergent loss of PTEN leads to clinical resistance to a PI(3)Kα inhibitor. Nature 518, 240–244. 10.1038/nature13948 25409150 PMC4326538

[B51] Juvekar A. Burga L. N. Hu H. Lunsford E. P. Ibrahim Y. H. Balmañà J. (2012). Combining a PI3K inhibitor with a PARP inhibitor provides an effective therapy for BRCA1-related breast cancer. Cancer Discov. 2, 1048–1063. 10.1158/2159-8290.CD-11-0336 22915751 PMC3733368

[B52] Kartal-Yandim M. Adan-Gokbulut A. Baran Y. (2016). Molecular mechanisms of drug resistance and its reversal in cancer. Crit. Rev. Biotechnol. 36, 716–726. 10.3109/07388551.2015.1015957 25757878

[B53] Kataoka Y. Mukohara T. Shimada H. Saijo N. Hirai M. Minami H. (2010). Association between gain-of-function mutations in PIK3CA and resistance to HER2-targeted agents in HER2-amplified breast cancer cell lines. Ann. Oncol. 21, 255–262. 10.1093/annonc/mdp304 19633047

[B54] Kathawala R. J. Gupta P. Ashby C. R. Jr. Chen Z. S. (2015). The modulation of ABC transporter-mediated multidrug resistance in cancer: a review of the past decade. Drug Resist. Updat 18, 1–17. 10.1016/j.drup.2014.11.002 25554624

[B55] Keegan N. M. Gleeson J. P. Hennessy B. T. Morris P. G. (2018). PI3K inhibition to overcome endocrine resistance in breast cancer. Expert Opin. Investig. Drugs 27, 1–15. 10.1080/13543784.2018.1417384 29252036

[B56] Kim S. B. Dent R. Im S. A. Espié M. Blau S. Tan A. R. (2017). Ipatasertib plus paclitaxel versus placebo plus paclitaxel as first-line therapy for metastatic triple-negative breast cancer (LOTUS): a multicentre, randomised, double-blind, placebo-controlled, phase 2 trial. Lancet Oncol. 18, 1360–1372. 10.1016/s1470-2045(17)30450-3 28800861 PMC5626630

[B57] Krop I. E. Mayer I. A. Ganju V. Dickler M. Johnston S. Morales S. (2016). Pictilisib for oestrogen receptor-positive, aromatase inhibitor-resistant, advanced or metastatic breast cancer (FERGI): a randomised, double-blind, placebo-controlled, phase 2 trial. Lancet Oncol. 17, 811–821. 10.1016/s1470-2045(16)00106-6 27155741 PMC5524539

[B58] Kulkarni A. Natarajan S. K. Chandrasekar V. Pandey P. R. Sengupta S. (2016). Combining immune checkpoint inhibitors and kinase-inhibiting supramolecular therapeutics for enhanced anticancer efficacy. ACS Nano 10, 9227–9242. 10.1021/acsnano.6b01600 27656909

[B59] Lazarevic V. Glimcher L. H. Lord G. M. (2013). T-bet: a bridge between innate and adaptive immunity. Nat. Rev. Immunol. 13, 777–789. 10.1038/nri3536 24113868 PMC6290922

[B60] Li J. Yen C. Liaw D. Podsypanina K. Bose S. Wang S. I. (1997). PTEN, a putative protein tyrosine phosphatase gene mutated in human brain, breast, and prostate cancer. Science 275, 1943–1947. 10.1126/science.275.5308.1943 9072974

[B61] Li X. Xu Y. Ding Y. Li C. Zhao H. Wang J. (2018). Posttranscriptional upregulation of HER3 by HER2 mRNA induces trastuzumab resistance in breast cancer. Mol. Cancer 17, 113. 10.1186/s12943-018-0862-5 30068375 PMC6090962

[B62] Liu L. Meng T. Zheng X. Liu Y. Hao R. Yan Y. (2019). Transgelin 2 promotes paclitaxel resistance, migration, and invasion of breast cancer by directly interacting with PTEN and activating PI3K/Akt/GSK-3β pathway. Mol. Cancer Ther. 18, 2457–2468. 10.1158/1535-7163.mct-19-0261 31488699

[B63] Liu P. Cheng H. Roberts T. M. Zhao J. J. (2009). Targeting the phosphoinositide 3-kinase pathway in cancer. Nat. Rev. Drug Discov. 8, 627–644. 10.1038/nrd2926 19644473 PMC3142564

[B64] Liu S. Tang Y. Yan M. Jiang W. (2018). PIK3CA mutation sensitizes breast cancer cells to synergistic therapy of PI3K inhibition and AMPK activation. Invest. New Drugs 36, 763–772. 10.1007/s10637-018-0563-3 29504069

[B65] Loibl S. de la Pena L. Nekljudova V. Zardavas D. Michiels S. Denkert C. (2017). Neoadjuvant buparlisib plus trastuzumab and paclitaxel for women with HER2+ primary breast cancer: a randomised, double-blind, placebo-controlled phase II trial (NeoPHOEBE). Eur. J. Cancer 85, 133–145. 10.1016/j.ejca.2017.08.020 28923573 PMC5640494

[B66] Luo J. Yao J. F. Deng X. F. Zheng X. D. Jia M. Wang Y. Q. (2018). 14, 15-EET induces breast cancer cell EMT and cisplatin resistance by up-regulating integrin αvβ3 and activating FAK/PI3K/AKT signaling. J. Exp. Clin. Cancer Res. 37, 23. 10.1186/s13046-018-0694-6 29426357 PMC5807756

[B67] Maira S. M. Pecchi S. Huang A. Burger M. Knapp M. Sterker D. (2012). Identification and characterization of NVP-BKM120, an orally available pan-class I PI3-kinase inhibitor. Mol. Cancer Ther. 11, 317–328. 10.1158/1535-7163.mct-11-0474 22188813

[B68] Mansouri S. Feizi N. Mahdi A. Majidzadeh A. K. Farahmand L. (2018). A review on the role of VEGF in tamoxifen resistance. Anticancer Agents Med. Chem. 18, 2006–2009. 10.2174/1871520618666180911142259 30207246

[B69] Markham A. (2019). Alpelisib: first global approval. Drugs 79, 1249–1253. 10.1007/s40265-019-01161-6 31256368

[B70] Marks D. K. Gartrell R. D. El Asmar M. Boboila S. Hart T. Lu Y. (2020). Akt inhibition is associated with favorable immune profile changes within the tumor microenvironment of hormone receptor positive, HER2 negative breast cancer. Front. Oncol. 10, 968. 10.3389/fonc.2020.00968 32612958 PMC7308467

[B71] Martín M. Chan A. Dirix L. O’Shaughnessy J. Hegg R. Manikhas A. (2017). A randomized adaptive phase II/III study of buparlisib, a pan-class I PI3K inhibitor, combined with paclitaxel for the treatment of HER2- advanced breast cancer (BELLE-4). Ann. Oncol. 28, 313–320. 10.1093/annonc/mdw562 27803006

[B72] Masoumi H. Soltani A. Ghatrehsamani M. (2020). The beneficial role of SIRT1 activator on chemo- and radiosensitization of breast cancer cells in response to IL-6. Mol. Biol. Rep. 47, 129–139. 10.1007/s11033-019-05114-w 31781916

[B73] Mayer I. A. Abramson V. G. Formisano L. Balko J. M. Estrada M. V. Sanders M. E. (2017). A phase Ib study of alpelisib (BYL719), a PI3kα-specific inhibitor, with letrozole in ER+/HER2- metastatic breast cancer. Clin. Cancer Res. 23, 26–34. 10.1158/1078-0432.ccr-16-0134 27126994 PMC5085926

[B74] Mayer I. A. Abramson V. G. Isakoff S. J. Forero A. Balko J. M. Kuba M. G. (2014). Stand up to cancer phase Ib study of pan-phosphoinositide-3-kinase inhibitor buparlisib with letrozole in estrogen receptor-positive/human epidermal growth factor receptor 2-negative metastatic breast cancer. J. Clin. Oncol. 32, 1202–1209. 10.1200/jco.2013.54.0518 24663045 PMC3986383

[B75] Michaloglou C. Crafter C. Siersbaek R. Delpuech O. Curwen J. O. Carnevalli L. S. (2018). Combined inhibition of mTOR and CDK4/6 is required for optimal blockade of E2F function and long-term growth inhibition in estrogen receptor-positive breast cancer. Mol. Cancer Ther. 17, 908–920. 10.1158/1535-7163.mct-17-0537 29483206 PMC6485624

[B76] Miller T. W. Balko J. M. Arteaga C. L. (2011a). Phosphatidylinositol 3-kinase and antiestrogen resistance in breast cancer. J. Clin. Oncol. 29, 4452–4461. 10.1200/jco.2010.34.4879 22010023 PMC3221526

[B77] Miller T. W. Balko J. M. Fox E. M. Ghazoui Z. Dunbier A. Anderson H. (2011c). ERα-dependent E2F transcription can mediate resistance to estrogen deprivation in human breast cancer. Cancer Discov. 1, 338–351. 10.1158/2159-8290.cd-11-0101 22049316 PMC3204388

[B78] Miller T. W. Hennessy B. T. González-Angulo A. M. Fox E. M. Mills G. B. Chen H. (2010). Hyperactivation of phosphatidylinositol-3 kinase promotes escape from hormone dependence in estrogen receptor-positive human breast cancer. J. Clin. Invest. 120, 2406–2413. 10.1172/jci41680 20530877 PMC2898598

[B79] Miller T. W. Rexer B. N. Garrett J. T. Arteaga C. L. (2011b). Mutations in the phosphatidylinositol 3-kinase pathway: role in tumor progression and therapeutic implications in breast cancer. Breast Cancer Res. 13, 224. 10.1186/bcr3039 22114931 PMC3315683

[B80] Mollon L. E. Anderson E. J. Dean J. L. Warholak T. L. Aizer A. Platt E. A. (2020). A systematic literature review of the prognostic and predictive value of PIK3CA mutations in HR(+)/HER2(−) metastatic breast cancer. Cancer Manag. Res. 20, e232–e243. 10.2147/cmar.s202965 32234362

[B81] Mukohara T. (2015). PI3K mutations in breast cancer: prognostic and therapeutic implications. Breast Cancer 7, 111–123. 10.2147/bctt.s60696 26028978 PMC4440424

[B82] Murphy C. G. (2019). The role of CDK4/6 inhibitors in breast cancer. Curr. Treat. Options. Oncol. 20, 483. 10.1007/s11864-019-0651-4 31101994

[B83] Nagini S. (2017). Breast cancer: current molecular therapeutic targets and new players. Anticancer Agents Med. Chem. 17, 152–163. 10.2174/1871520616666160502122724 27137076

[B84] Ndubaku C. O. Heffron T. P. Staben S. T. Baumgardner M. Blaquiere N. Bradley E. (2013). Discovery of 2-{3-[2-(1-isopropyl-3-methyl-1H-1,2-4-triazol-5-yl)-5,6-dihydrobenzo[f]imidazo[1,2-d][1,4]oxazepin-9-yl]-1H-pyrazol-1-yl}-2-methylpropanamide (GDC-0032): a β-sparing phosphoinositide 3-kinase inhibitor with high unbound exposure and robust *in vivo* antitumor activity. J. Med. Chem. 56, 4597–4610. 10.1021/jm4003632 23662903

[B85] Network C. G. A. (2012). Comprehensive molecular portraits of human breast tumours. Nature 490, 61–70. 10.1038/nature11412 23000897 PMC3465532

[B86] No M. Choi E. J. Kim I. A. (2009). Targeting HER2 signaling pathway for radiosensitization: alternative strategy for therapeutic resistance. Cancer Biol. Ther. 8, 2351–2361. 10.4161/cbt.8.24.10131 19923913

[B87] O’Leary B. Cutts R. J. Liu Y. Hrebien S. Huang X. Fenwick K. (2018). The genetic landscape and clonal evolution of breast cancer resistance to palbociclib plus fulvestrant in the PALOMA-3 trial. Cancer Discov. 8, 1390–1403. 10.1158/2159-8290.cd-18-0264 30206110 PMC6368247

[B88] Olgen S. (2018). Overview on anticancer drug design and development. Curr. Med. Chem. 25, 1704–1719. 10.2174/0929867325666171129215610 29189124

[B89] Owusu-Brackett N. Zhao M. Akcakanat A. Evans K. W. Yuca E. Dumbrava E. I. (2020). Targeting PI3Kβ alone and in combination with chemotherapy or immunotherapy in tumors with PTEN loss. Oncotarget 11, 969–981. 10.18632/oncotarget.27503 32215185 PMC7082117

[B90] O’Brien C. Wallin J. J. Sampath D. GuhaThakurta D. Savage H. Punnoose E. A. (2010). Predictive biomarkers of sensitivity to the phosphatidylinositol 3’ kinase inhibitor GDC-0941 in breast cancer preclinical models. Clin. Cancer Res. 16, 3670–3683. 10.1158/1078-0432.ccr-09-2828 20453058

[B91] O’Brien N. A. McDonald K. Tong L. von Euw E. Kalous O. Conklin D. (2014). Targeting PI3K/mTOR overcomes resistance to HER2-targeted therapy independent of feedback activation of AKT. Clin. Cancer Res. 20, 3507–3520. 10.1158/1078-0432.ccr-13-2769 24879796

[B92] Patton D. T. Garden O. A. Pearce W. P. Clough L. E. Monk C. R. Leung E. (2006). Cutting edge: the phosphoinositide 3-kinase p110 delta is critical for the function of CD4+CD25+Foxp3+ regulatory T cells. J. Immunol. 177, 6598–6602. 10.4049/jimmunol.177.10.6598 17082571

[B93] Pernas S. Tolaney S. M. (2019). HER2-positive breast cancer: new therapeutic frontiers and overcoming resistance. Ther. Adv. Med. Oncol. 11, 1758835919833519. 10.1177/1758835919833519 30911337 PMC6425535

[B94] Pogue-Geile K. L. Song N. Jeong J. H. Gavin P. G. Kim S. R. Blackmon N. L. (2015). Intrinsic subtypes, PIK3CA mutation, and the degree of benefit from adjuvant trastuzumab in the NSABP B-31 trial. J. Clin. Oncol. 33, 1340–1347. 10.1200/jco.2014.56.2439 25559813 PMC4397278

[B95] Qiu N. He Y. F. Zhang S. M. Zhan Y. T. Han G. D. Jiang M. (2019). Cullin7 enhances resistance to trastuzumab therapy in Her2 positive breast cancer via degrading IRS-1 and downregulating IGFBP-3 to activate the PI3K/AKT pathway. Cancer Lett. 464, 25–36. 10.1016/j.canlet.2019.08.008 31461670

[B96] Ramirez-Ardila D. E. Helmijr J. C. Look M. P. Lurkin I. Ruigrok-Ritstier K. van Laere S. (2013). Hotspot mutations in PIK3CA associate with first-line treatment outcome for aromatase inhibitors but not for tamoxifen. Breast Cancer Res. Treat. 139, 39–49. 10.1007/s10549-013-2529-7 23592373

[B97] Razis E. Bobos M. Kotoula V. Eleftheraki A. G. Kalofonos H. P. Pavlakis K. (2011). Evaluation of the association of PIK3CA mutations and PTEN loss with efficacy of trastuzumab therapy in metastatic breast cancer. Breast Cancer Res. Treat. 128, 447–456. 10.1007/s10549-011-1572-5 21594665

[B98] Sabine V. S. Crozier C. Brookes C. L. Drake C. Piper T. van de Velde C. J. (2014). Mutational analysis of PI3K/AKT signaling pathway in tamoxifen exemestane adjuvant multinational pathology study. J. Clin. Oncol. 32, 2951–2958. 10.1200/jco.2013.53.8272 25071141

[B99] Sanchez C. G. Ma C. X. Crowder R. J. Guintoli T. Phommaly C. Gao F. (2011). Preclinical modeling of combined phosphatidylinositol-3-kinase inhibition with endocrine therapy for estrogen receptor-positive breast cancer. Breast Cancer Res. 13, R21. 10.1186/bcr2833 21362200 PMC3219179

[B100] Schmid P. Pinder S. E. Wheatley D. Macaskill J. Zammit C. Hu J. (2016). Phase II randomized preoperative window-of-opportunity study of the PI3K inhibitor pictilisib plus anastrozole compared with anastrozole alone in patients with estrogen receptor-positive breast cancer. J. Clin. Oncol. 34, 1987–1994. 10.1200/jco.2015.63.9179 26976426 PMC6075966

[B101] Schötz U. Balzer V. Brandt F. W. Ziemann F. Subtil F. S. B. Rieckmann T. (2020). Dual PI3K/mTOR inhibitor NVP-BEZ235 enhances radiosensitivity of head and neck squamous cell carcinoma (HNSCC) cell lines due to suppressed double-strand break (DSB) repair by non-homologous end joining. Cancers 12 (2), 467. 10.3390/cancers12020467 PMC7072694 32085396

[B102] Serra V. Eichhorn P. J. García-García C. Ibrahim Y. H. Prudkin L. Sánchez G. (2013). RSK3/4 mediate resistance to PI3K pathway inhibitors in breast cancer. J. Clin. Invest. 123, 2551–2563. 10.1172/jci66343 23635776 PMC3668839

[B103] Siegel R. L. Miller K. D. (2020). Cancer statistics. CA Cancer J. Clin. 70, 7–30. 10.3322/caac.21590 31912902

[B104] Soare G. R. Soare C. A. (2019). Immunotherapy for breast cancer: first FDA approved regimen. Discoveries 7, e91. 10.15190/d.2019.4 32309609 PMC7086078

[B105] Stern H. M. Gardner H. Burzykowski T. Elatre W. O’Brien C. Lackner M. R. (2015). PTEN loss is associated with worse outcome in HER2-amplified breast cancer patients but is not associated with trastuzumab resistance. Clin. Cancer Res. 21, 2065–2074. 10.1158/1078-0432.ccr-14-2993 25649019 PMC4417419

[B106] Sun C. K. Zhang F. Xiang T. Chen Q. Pandita T. K. Huang Y. (2014). Phosphorylation of ribosomal protein S6 confers PARP inhibitor resistance in BRCA1-deficient cancers. Oncotarget 5, 3375–3385. 10.18632/oncotarget.1952 24831086 PMC4102816

[B107] Tapodi A. Bognar Z. Szabo C. Gallyas F. Sumegi B. Hocsak E. (2019). PARP inhibition induces Akt-mediated cytoprotective effects through the formation of a mitochondria-targeted phospho-ATM-NEMO-Akt-mTOR signalosome. Biochem. Pharmacol. 162, 98–108. 10.1016/j.bcp.2018.10.005 30296409

[B108] Tapodi A. Debreceni B. Hanto K. Bognar Z. Wittmann I. Gallyas F. Jr. (2005). Pivotal role of Akt activation in mitochondrial protection and cell survival by poly(ADP-ribose)polymerase-1 inhibition in oxidative stress. J. Biol. Chem. 280, 35767–35775. 10.1074/jbc.M507075200 16115861

[B109] Tazzari P. L. Cappellini A. Ricci F. Evangelisti C. Papa V. Grafone T. (2007). Multidrug resistance-associated protein 1 expression is under the control of the phosphoinositide 3 kinase/Akt signal transduction network in human acute myelogenous leukemia blasts. Leukemia 21, 427–438. 10.1038/sj.leu.2404523 17215852

[B110] Tompa R. (2018). First PARP inhibitor ok’d for breast cancer. Cancer Discov. 8, 256–257. 10.1158/2159-8290.cd-nb2018-008 29382645

[B111] Tsou S. H. Chen T. M. Hsiao H. T. Chen Y. H. (2015). A critical dose of doxorubicin is required to alter the gene expression profiles in MCF-7 cells acquiring multidrug resistance. PLoS One 10, e0116747. 10.1371/journal.pone.0116747 25635866 PMC4312059

[B112] van der Waart A. B. van de Weem N. M. Maas F. Kramer C. S. Kester M. G. Falkenburg J. H. (2014). Inhibition of Akt signaling promotes the generation of superior tumor-reactive T cells for adoptive immunotherapy. Blood 124, 3490–3500. 10.1182/blood-2014-05-578583 25336630 PMC4246043

[B113] Veres B. Gallyas F. Jr. Varbiro G. Berente Z. Osz E. Szekeres G. (2003). Decrease of the inflammatory response and induction of the Akt/protein kinase B pathway by poly-(ADP-ribose) polymerase 1 inhibitor in endotoxin-induced septic shock. Biochem. Pharmacol. 65, 1373–1382. 10.1016/s0006-2952(03)00077-7 12694878

[B114] Veres B. Radnai B. Gallyas F. Jr. Varbiro G. Berente Z. Osz E. (2004). Regulation of kinase cascades and transcription factors by a poly(ADP-ribose) polymerase-1 inhibitor, 4-hydroxyquinazoline, in lipopolysaccharide-induced inflammation in mice. J. Pharmacol. Exp. Ther. 310, 247–255. 10.1124/jpet.104.065151 14999056

[B115] Verret B. Cortes J. Bachelot T. Andre F. Arnedos M. (2019). Efficacy of PI3K inhibitors in advanced breast cancer. Ann. Oncol. 30 (Suppl. 10), x12–x20. 10.1093/annonc/mdz381 31928690

[B116] Vora S. R. Juric D. Kim N. Mino-Kenudson M. Huynh T. Costa C. (2014). CDK 4/6 inhibitors sensitize PIK3CA mutant breast cancer to PI3K inhibitors. Cancer Cell 26, 136–149. 10.1016/j.ccr.2014.05.020 25002028 PMC4155598

[B117] Vuylsteke P. Huizing M. Petrakova K. Roylance R. Laing R. Chan S. (2016). Pictilisib PI3Kinase inhibitor (a phosphatidylinositol 3-kinase [PI3K] inhibitor) plus paclitaxel for the treatment of hormone receptor-positive, HER2-negative, locally recurrent, or metastatic breast cancer: interim analysis of the multicentre, placebo-controlled, phase II randomised PEGGY study. Ann. Oncol. 27, 2059–2066. 10.1093/annonc/mdw320 27573562

[B118] Wang H. Jia X. H. Chen J. R. Wang J. Y. Li Y. J. (2016a). Osthole shows the potential to overcome P-glycoprotein-mediated multidrug resistance in human myelogenous leukemia K562/ADM cells by inhibiting the PI3K/Akt signaling pathway. Oncol. Rep. 35, 3659–3668. 10.3892/or.2016.4730 27109742

[B119] Wang Q. Liu P. Spangle J. M. Von T. Roberts T. M. Lin N. U. (2016b). PI3K-p110α mediates resistance to HER2-targeted therapy in HER2+, PTEN-deficient breast cancers. Oncogene 35, 3607–3612. 10.1038/onc.2015.406 26500061 PMC4846581

[B120] Wanigasooriya K. Tyler R. Barros-Silva J. D. Sinha Y. Ismail T. Beggs A. D. (2020). Radiosensitising cancer using phosphatidylinositol-3-kinase (PI3K), protein kinase B (AKT) or mammalian target of rapamycin (mTOR) inhibitors. Cancers 12 (5), 1278. 10.3390/cancers12051278 PMC7281073 32443649

[B121] Wee S. Wiederschain D. Maira S. M. Loo A. Miller C. deBeaumont R. (2008). PTEN-deficient cancers depend on PIK3CB. Proc. Natl. Acad. Sci. U.S.A. 105, 13057–13062. 10.1073/pnas.0802655105 18755892 PMC2529105

[B122] Wolff A. C. Lazar A. A. Bondarenko I. Garin A. M. Brincat S. Chow L. (2013). Randomized phase III placebo-controlled trial of letrozole plus oral temsirolimus as first-line endocrine therapy in postmenopausal women with locally advanced or metastatic breast cancer. J. Clin. Oncol. 31, 195–202. 10.1200/jco.2011.38.3331 23233719 PMC3532391

[B123] Xi J. Ma C. X. (2020). Sequencing endocrine therapy for metastatic breast cancer: what do we do after disease progression on a CDK4/6 inhibitor? Curr. Oncol. Rep. 22, 57. 10.1007/s11912-020-00917-8 32415339

[B124] Yap T. A. Bjerke L. Clarke P. A. Workman P. (2015). Drugging PI3K in cancer: refining targets and therapeutic strategies. Curr. Opin. Pharmacol. 23, 98–107. 10.1016/j.coph.2015.05.016 26117819 PMC4728196

[B125] Yardley D. A. Noguchi S. Pritchard K. I. Burris H. A. 3rd Baselga J. Gnant M. (2013). Everolimus plus exemestane in postmenopausal patients with HR(+) breast cancer: BOLERO-2 final progression-free survival analysis. Adv. Ther. 30, 870–884. 10.1007/s12325-013-0060-1 24158787 PMC3898123

[B126] Yu C. C. Hung S. K. Lin H. Y. Chiou W. Y. Lee M. S. Liao H. F. (2017). Targeting the PI3K/AKT/mTOR signaling pathway as an effectively radiosensitizing strategy for treating human oral squamous cell carcinoma *in vitro* and *in vivo* . Oncotarget 8, 68641–68653. 10.18632/oncotarget.19817 28978144 PMC5620284

[B127] Zhang C. Xu B. Liu P. (2016a). Addition of the p110α inhibitor BYL719 overcomes targeted therapy resistance in cells from Her2-positive-PTEN-loss breast cancer. Tumour Biol. 37, 14831–14839. 10.1007/s13277-016-5381-7 27639383

[B128] Zhang H. Yu N. Chen Y. Yan K. Wang X. (2019). Cationic liposome codelivering PI3K pathway regulator improves the response of BRCA1-deficient breast cancer cells to PARP1 inhibition. J. Cell Biochem 120, 13037–13045. 10.1002/jcb.28574 30873673

[B129] Zhang J. Xu K. Liu P. Geng Y. Wang B. Gan W. (2016b). Inhibition of Rb phosphorylation leads to mTORC2-mediated activation of akt. Mol. Cell 62, 929–942. 10.1016/j.molcel.2016.04.023 27237051 PMC4912424

[B130] Zhang L. Li Y. Wang Q. Chen Z. Li X. Wu Z. (2020). The PI3K subunits, P110α and P110β are potential targets for overcoming P-gp and BCRP-mediated MDR in cancer. Mol. Cancer 19, 10. 10.1186/s12943-019-1112-1 31952518 PMC6966863

[B131] Zhao M. Ramaswamy B. (2014). Mechanisms and therapeutic advances in the management of endocrine-resistant breast cancer. World J. Clin. Oncol. 5, 248–262. 10.5306/wjco.v5.i3.248 25114842 PMC4127598

[B132] Zhong J. T. Yu J. Wang H. J. Shi Y. Zhao T. S. He B. X. (2017). Effects of endoplasmic reticulum stress on the autophagy, apoptosis, and chemotherapy resistance of human breast cancer cells by regulating the PI3K/AKT/mTOR signaling pathway. Tumour Biol. 39 (5), 1010428317697562. 10.1177/1010428317697562 28459209

[B133] Zou Z. Zhang J. Zhang H. Liu H. Li Z. Cheng D. (2014). 3-Methyladenine can depress drug efflux transporters via blocking the PI3K-AKT-mTOR pathway thus sensitizing MDR cancer to chemotherapy. J. Drug Target. 22, 839–848. 10.3109/1061186x.2014.936870 25019701

